# Machine-learning-based identification of patients with IgA nephropathy using a computerized medical billing database

**DOI:** 10.1371/journal.pone.0312915

**Published:** 2024-12-05

**Authors:** Ryoya Tsunoda, Keitaro Kume, Rina Kagawa, Masaru Sanuki, Hiroyuki Kitagawa, Kaori Mase, Kunihiro Yamagata

**Affiliations:** 1 Faculty of Medicine, Department of Nephrology, University of Tsukuba, Tsukuba, Japan; 2 Faculty of Medicine, Department of Clinical Medicine, University of Tsukuba, Tsukuba, Japan; 3 Faculty of Medicine, Department of Biomedical Informatics and Management, University of Tsukuba, Tsukuba, Japan; 4 International Institute for Integrative Sleep Medicine, University of Tsukuba, Tsukuba, Japan; Vellore Institute of Technology, INDIA

## Abstract

The billing database of the universal healthcare system in Japan potentially includes large-cohort data of patients with immunoglobulin A nephropathy, diagnosis codes aimed at billing should not be directly used for clinical research because of the risk of misdiagnosis. To solve this problem, we aimed to develop a novel method for identifying patients with immunoglobulin A nephropathy from billing data using machine learning. The medical records and bills of 3,743 patients who consulted nephrologists at a single center were extracted. Patients were labeled to have been diagnosed with immunoglobulin A nephropathy through a review of medical records. A manual analysis of the diagnostic accuracy and machine learning was performed. For machine learning, the datasets were preprocessed in three patterns and assigned to the XGBoost program using five-fold cross-validation. Of all the participants, 437 were labeled as having been diagnosed with immunoglobulin A nephropathy. Bill codes for immunoglobulin A nephropathy were provided to approximately half of them. The manually created criteria consisting of the recommended examinations and treatments in the Japanese guidelines for immunoglobulin A nephropathy showed both specificity and sensitivity < 0.8. In contrast, with the receiver operating characteristic curve analysis, the machine learning process yielded area under the curve values over 0.9 with preprocessing from the clinical viewpoint. Applying machine learning technology to a dataset preprocessed from a clinical viewpoint achieved a high performance in detecting patients with immunoglobulin A nephropathy. This methodology contributes to the construction of a disease-specific cohort using big bill data.

## Introduction

Immunoglobulin A (IgA) nephropathy (IgAN) is the most common type of glomerulonephritis [1]. The incidence of IgAN varies by region, with an annual incidence (per 100,000 population) of 0.76–2.5 in Europe [2,3] and 4.5 in Japan [4]; although the estimated point prevalence is 2.53 per 10,000 in Europe [3], it may be higher in Japan. The pathogenesis of IgAN warrants long-term observations, over decades, to evaluate the effects of different treatments on renal outcomes [5,6]. Thus, there is a strong need for large clinical studies to determine the treatment efficacy based on long-term observations.

Under the universal healthcare system, the National Database of Health Insurance Claims and Specific Health Checkups of Japan (NDB) contains the billing data of almost all citizens in Japan [7]. Although this database has been used for research earlier, the majority were descriptive or cross-sectional studies [8] because of the paucity of clinical information on the diseases of patients. Thus, using this approach, it is difficult to estimate the survival of patients.

In contrast, end-stage kidney disease is almost always fully recorded on the bill at the time of initiation of dialysis or kidney transplantation. Thus, identifying patients with specific kidney diseases, such as IgAN, from the billing database might be an innovative method of creating the largest IgAN database that includes the complete implementation record of examinations and treatments. However, a critical barrier to the extraction of specific diagnostic information using billing data is that the diagnoses recorded on medical bills do not necessarily correspond to the patients’ actual clinical conditions because the diagnostic codes have been given only for reward claims. Correct diagnoses cannot be obtained using diagnostic codes alone, and this issue has been persistently reported by researchers [9]. In the field of nephrology, the coding of CKD and acute kidney disease has low sensitivity for a true clinical diagnosis [10]. Moreover, the inaccurate diagnostic codes in billing data have been reported for many diseases, including schizophrenia [11], hypertension [12], diabetes mellitus [13], rheumatological diseases [14], and cardiovascular events [15]. Thus, there exists a serious risk of misdiagnosis when only diagnostic codes are used to identify patients with any specific disease.

There are two types of biases in the diagnostic codes for medical bills. One bias is “underdiagnosis.” In other words, an important diagnosis that does not pertain to the claim for payment is not necessarily included in bills. In most patients who consult a nephrologist for the treatment of IgAN, a diagnosis of “CKD” or “chronic glomerulonephritis” is sufficient for an appropriate claim because there is no test or treatment specific for IgAN. It is difficult to correctly detect patients with IgAN using codes alone.

Another bias is “overdiagnosis.” It is possible that the given diagnostic codes do not fully match the patient’s clinical condition. For example, patients diagnosed with hypertension “hypertension.” In Japan, angiotensin II receptor blockers, the recommended drugs for IgAN, are approved by the Pharmaceuticals and Medical Devices Agency and MHLW only for the treatment of hypertension or diabetic nephropathy. Therefore, when a patient with IgAN presents with slightly high blood pressure that is not diagnosed as hypertension under regular practice, clinicians may diagnose it as hypertension based on the patient’s medical bill. Moreover, diagnostic codes for IgAN do not necessarily indicate true IgAN because they are not always based on biopsy results. Therefore, the enrollment of patients based solely on the diagnoses recorded on medical bills in clinical research is risky.

In some areas, there have been few reports on the detection of clinical diagnoses from bill data. One such example is type 1 diabetes mellitus (T1DM). Okui et al. reported that patients with T1DM were successfully classified using the bills data [16]. However, most T1DM patients require insulin infusions. In other words, non-use of insulin is a powerful factor in excluding T1DM. Currently, there is no specific treatment for IgAN. This implies that achieving the goal in a manner similar to that in the previous report is impossible.

Several studies have adapted machine learning to big data to identify patients with specific diseases. For example, Nasibeh et al. reported the merits of applying random forest to electronic health record data to detect patients with hypertrophic cardiomyopathy [17]. A random forest was used to produce a predictive model for the clinical diagnosis of hypertrophic cardiomyopathy in each patient using health record data. Machine learning is a form of artificial intelligence that enables computers to recognize inputs and decide what to do to achieve specific goal [18]. Qin et al. reported that patients with CKD with missing clinical data were detected with an accuracy of more than 99% using machine learning technology [19]. Additionally, several studies have developed predictive models for kidney disease using clinical data, which are assumed to be useful for detecting CKD [20–23]. Koyner et al. reported that machine learning had a high performance in predicting acute kidney injury before an increase in serum creatinine levels [24].

However, the aim of these studies was to utilize machine learning as a tool to help clinicians make decisions, and few studies have reported its influence on clinical treatment [25]. To the best of our knowledge, no study has applied machine learning to bill data to detect patients who have already been diagnosed with a specific disease to construct a disease-specific database available for clinical research.

Therefore, we focused on a large public medical insurance system database derived from the nationwide universal healthcare system in Japan. In this study, we aimed to develop a novel method for determining whether an IgAN diagnosis was obtained exclusively from billing data, which were assumed to be utilized for the creation of the disease-specific IgAN cohort from a billing database.

## Methods

### Data source

**Fig 1:**
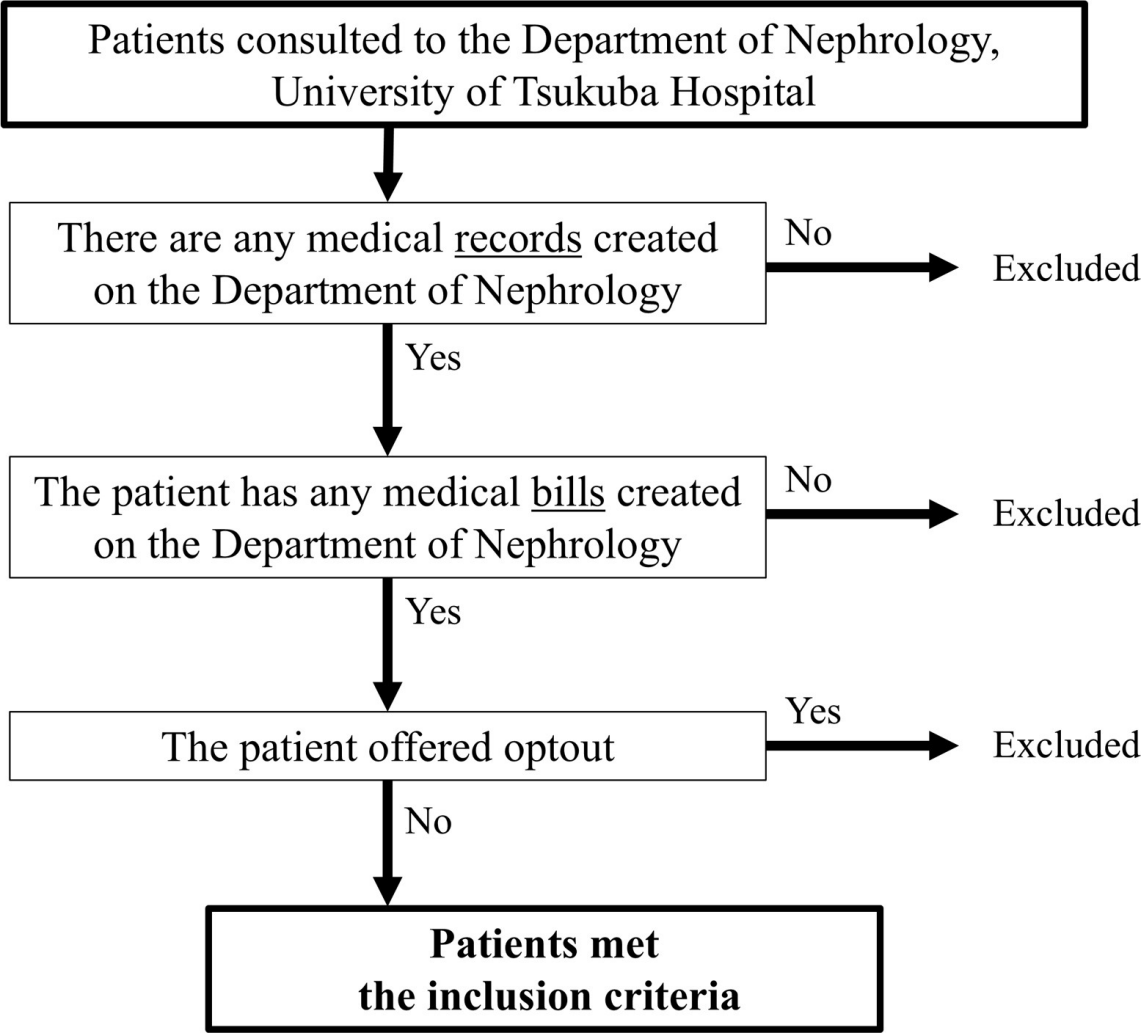
Flowchart of the recruitment, inclusion, and exclusion of data sources.

This descriptive analytical study enrolled patients from the Department of Nephrology at the University of Tsukuba Hospital. Participants were patients who consulted the department at least once between January 1, 2013, and July 31, 2019. The inclusion and exclusion criteria are shown in **Fig 1**.

### Data extraction

All medical records, including the daily charts and medical bills of the participants, were extracted from the data server of the hospital’s medical ordering system. Billing data included the name of the hospital that generated the bill, the type of insurance the patient used, diagnostic codes for billing, the itemization of the examinations and treatments that the patients received, and an explanation of the reason for any discrepancy between the examinations or treatments and diagnostic codes of each patient.

All collected data were anonymized and virtual IDs were provided to all patients. All data were transformed into numerical forms that indicated the number of times each claim code appeared, except for the binomial form of the presence of the diagnostic codes, during the entire observation period.

Medications were recorded in the form of codes that could only be processed in the electronic bill system, which otherwise had no meaning. These codes were converted into Japanese universal codes provided by the MHLW, known as “MHLW codes” that have regularity in the type of effect of medicines to make it possible to classify patients in clinical characteristics. For example, code 2171005F2021 shows the medicine “COMELIAN KOWA Tablets 100 mg,” a brand of dilazep hydrochloride hydrate. The first 4 digits (“2171”) represent the classification of coronary artery dilators, including the first upper classification of 3 digits (“217,” the vasodilator). The next 3 digits (“005”) represent the specific compound of the classification (dilazep hydrochloride hydrate) with the classification of administration (oral: 000–399; infusion: 400–699; or external: 700–999). The letter next to 7 digits (“F”) represents the form of the medicine (tablet form). The digit next to the letter (“2”) represents the dose of a singular unit (100 mg). The last 3 digits (“021”) represent the unique product of the same form of the same compound (“COMELIAN KOWA”). Using this code, medicines can be classified in specific ways, such as their effects, specific compounds, or specific products.

### Review of medical records for the classification of patients

All participants were diagnosed with IgAN during the study period based on a review of their medical records. The diagnosis of IgAN was confirmed when the patient met at least one of the following criteria: 1) description of the presence of diagnosed IgAN in the medical records; 2) diagnosis of IgAN on the pathology report of kidney biopsy; and 3) diagnosis of IgAN on a medical letter from another hospital. Based on this assessment process, specific labels were developed for the presence of IgAN in the patients.

### Analysis

Analysis was performed using two methods: manual analysis and machine learning. These two methods were compared to investigate which approach was better for detecting patients with IgAN from medical bill data.

#### Manual analysis

Some arbitrary criteria were created by using billing data, based on diagnosis and standard therapy, according to the clinical guidelines and opinions regarding IgAN. The criteria for this process included diagnostic codes, examinations, and treatment of the patients.

The data used for diagnosis and treatment were shaped according to the definitions listed in **Table 1**. For each of the criteria and their combination, the sensitivity, specificity, positive predictive value (PV+), negative predictive value (PV−), and accuracy for each criterion for the detection of IgAN were analyzed.

**Table 1:** Characteristics of objects.

	Definition of codes*	IgAN	Non-IgAN	p
**Numbers of patients**	-	437	3,306	
**Age at the point of the oldest data**	-	47.1 ± 16.1	57.1 ± 25.1	<0.001
**Female**	-	205 (46.9%)	1,409 (42.6%)	0.1992
**Presence of the diagnostic code of**	-			
**Any glomerular disease**	ICD-10 N00x-N08x	380	1327	<0.001
acute nephritis syndrome	ICD-10 N00x	1	16	0.457
rapidly progressive glomerulonephritis	ICD-10 N01x	27	160	0.2393
recurrent or continuous hematuria	ICD-10 N02x	235	158	<0.0001
IgA nephropathy	ICD-10 N028	231	106	<0.001
chronic glomerulonephritis	ICD-10 N03x	275	718	<0.0001
nephrotic syndrome	ICD-10 N04x	108	561	<0.001
nephritis syndrome, not specified	ICD-10 N05x	31	203	0.4537
proteinuria with specified morphological change	ICD-10 N06x	0	0	
any type of mesangial proliferative glomerulonephritis	ICD-10 N033/N043/N053	1	3	0.4066
genetic nephropathy, not otherwise specified	ICD-10 N07x	0	4	0.4671
glomerular disorders, not otherwise specified	ICD-10 N08x	0	0	
acute renal failure	ICD-10 N17x	11	133	0.1315
chronic kidney diseases	ICD-10 N18x	133	1353	0.0011
renal failure, details unknown	ICD-10 N19x	100	1052	0.0016
**Presence of the billing code of serum IgA measurement**	Billing code of D015	276	1611	<0.001
only once		85	1049	<0.001
two times or more		191	562	<0.001
Code of percutaneous needle biopsy (does not necessarily imply kidney biopsy)	Billing code of D412	162	385	<0.001
**Code of immunostaining or immunofluorescence**	Billing code of N002	160	382	<0.001
**Use of any kind of renin–angiotensin system inhibitors:** • Angiotensin II-converting enzyme inhibitors or • angiotensin II receptor blockers or direct renin inhibitors)	MHLW codes of • 2144xxxxxxx • 2149xxxxxxx	293	1353	<0.001
**Use of corticosteroids**				
injectable	MHLW code of 2456xxxDxxx	60	151	<0.001
injectable or oral	MHLW code of 2456xxxxxxx	144	552	<0.001
**Use of specific coronary artery dilators (including dilazep)**	MHLW code of 2171xxxDxxx	238	1249	<0.001
**Treatment of tonsillitis**	Billing code of J098	2	0	<0.001

* The letter “*x*” in the column “Definition of codes” shows that the wildcard includes digits or letters.

In addition, scoring criteria for identifying patients with IgAN in the bill data were manually created. Five factors were selected according to the headings of the components of the Japanese guidelines [26] that are generally considered important in patients with IgAN.

1. Two or more serum IgA measurements
2. Needle biopsy with immunofluorescence or immunostaining
3. Use of angiotensin-converting enzymes inhibitors, direct renin inhibitors, or angiotensin II receptor blockers
4. Presence of a group of drugs that includes dilazep hydrochloride hydrate
5. Use of any form of corticosteroids
6. Treatment of tonsillitis

Based on the number of participants who fulfilled these criteria, ROC analyses were performed.

#### Machine learning

The data were analyzed using machine learning. Three datasets were preprocessed using three different methods.

- Raw data: medication and diagnostic codes were not compiled. Bill data were provided directly to the learning program after minimal cleaning, reshaping to a format suitable for the learning program, and removing possible leaky variables.
- Analyzed data with compiled codes of medication and diagnosis, except for diagnostic codes in nephrology: After preprocessing in the same way as in (a), medication and diagnostic codes were compiled in some regularity. Drugs with the same top four digits in the MHLW codes and the same route of administration were placed in the same type of drug. Diagnostic codes recorded as ICD-10 with the same top two letters were compiled into the same diagnosis group. Diagnostic codes in the nephrology category (N0xx–N2xx) were excluded.
- Analyzed data with compiled codes of medication and all diagnoses. In addition to (b), diagnostic codes in nephrology were compiled in the first three letters of the ICD-10.

For the machine-learning processes, the extreme gradient boosting (XGboost) method [27] was selected as the supervised learning method to construct classifiers for the presence of IgAN. There were three reasons for this selection. The first was the size of the dataset, with a maximum number of 3,743 patients, which is considered relatively small for machine learning and does not require excessive machine power. This implies that the fastest processing speed of LightGBM is not required. Second, the data source was a single center with a relatively small sample size. The risk of overfitting is not negligible and should be minimized. The LightGBM creates decision trees with fewer horizontal branches and a deeper hierarchy. According to this characteristic, the risk of overfitting–the excessive adaptation to unimportant details of the learning data–is a concern for LightGBM. Third, from many reports, it has been proved that XGboost practices are useful. Huang et al. reported that, compared to random forest, artificial neural networks, and adaptive boosting, XGboost performed best for predicting heart disease [28]. Moreover, XGBoost has demonstrated high predictive performance for identifying CKD in a clinical database [20]. CatBoost is the newest method that creates many decision trees with numerous patterns of variable weighting [27]; however, there are relatively few reports of this method.

To avoid over- and undertraining, an analysis was conducted using a 5-fold cross-validation method. In each pattern of compilation of diagnostic nephrology codes, as mentioned above, 3,743 patients were divided into five small groups with an equal number (748 or 749) of patients and an equal rate of IgAN patients (nearly 11.7%). Five patterns of allocation of labeled data and data for verification were defined. In each pattern, the representative group was set as the data used for verification, and the other four groups were set as labeled data used for the training process. Thus, each trial of applying XGboost to four groups and verifying it using the remaining group was performed in five patterns. The hyperparameters were set using Optuna–an automated algorism for detecting the optimal tuning of hyperparameters [29]. The representative optimal hyperparameters produced by Optuna are shown in **S1 Table**.

#### Evaluation of diagnostic performance

A receiver operating characteristic (ROC) curve for each manual scoring and machine learning result was constructed, and the area under the curve (AUC) was calculated.

#### Software

ROC curve analysis was performed using R x86_64-redhat-linux-gnu, version 3.6.0 (including “roc” and “roc.test” function in “pROC” package). Machine learning was performed on the environment of Python version 3.6.8, using “xgboost” library version 1.2.0 and “optuna” library version 2.9.1.

#### Statistical analysis

The significance of the difference in each characteristic variable between patients with and without IgAN is shown by *p*-value that was calculated using the chi-square test. The aggregated results of the means and standard deviations (SD) are presented as the mean ± SD.

### Ethical approval

This study was approved by the Ethics Committee for Clinical Research at the University of Tsukuba Hospital (approval number: R01-170), and an opt-out announcement posted at the hospital was used to enable patients to opt out of the study if they did not wish to participate.

## Results

### Overview of the data

**Fig 2:**
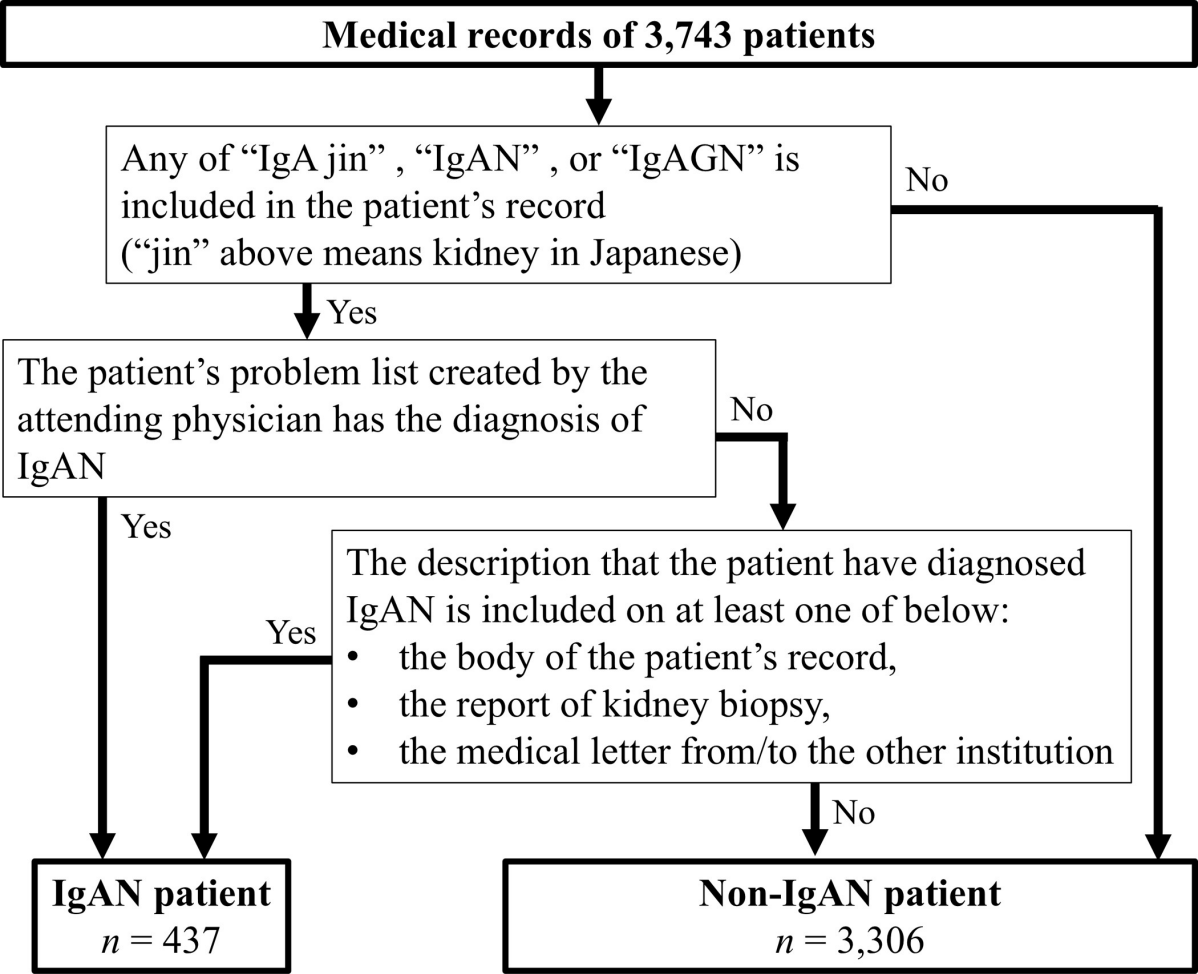
Flowchart depicting the algorism for determining whether a patient has IgAN, and the final number of patients confirmed to have or not have IgAN from the review of 3,743 medical records.

A total of 3,743 patients met the eligibility criteria shown in **Fig 1**. An overview of these data is presented in **Table 1**. As shown in **Fig 2**, among the 3,743 subjects, 437 (11.7%) were diagnosed with IgAN. Diagnostic codes for IgAN were provided for only 52.9% and 3.2% of patients with and without IgAN, respectively. Sensitivity, specificity, PV+, PV− and accuracy for single and created combined criteria are shown in **Table 2**. The PV+ value of the IgAN diagnostic codes was 68.5%. Among the patients with IgAN, 13.0% were not assigned any diagnostic codes for glomerular diseases. Moreover, 293 (67.0% of patients with IgAN), 144 (33.0%), and 238 (54.5%) patients received renin-angiotensin system inhibitors, corticosteroids, and a category of vasodilatory agents (including dilazeps), respectively.

**Table 2:** Results of manual analysis for test performance that included factors related to the IgAN diagnosis and guideline-recommended treatments.

	Sn	Sp	PV+	PV-	Ac
**Diagnostic components**					
1) Codes of IgAN	0.5286	0.9679	0.6855	0.9395	0.9166
2) Codes of recurrent hematuria or chronic glomerulonephritis (N02x/N03x)	0.8009	0.7632	0.3089	0.9667	0.7676
3) Any code of glomerular diseases (N00x–N08x)	0.8696	0.5986	0.2226	0.9720	0.6302
4) Measurement of serum IgA	0.6316	0.5127	0.1463	0.9133	0.5266
5) Measurement of serum IgA at least twice	0.4371	0.8300	0.2537	0.9177	0.7841
6) Needle biopsy with immunostaining/immunofluorescence	0.3570	0.8935	0.3071	0.9131	0.8309
**Treatment components**					
a) Specific coronary artery dilators (including dilazep)	0.5446	0.6222	0.1601	0.9118	0.6131
b) Renin–angiotensin inhibitors	0.6705	0.5907	0.1780	0.9313	0.6001
c) Corticosteroids in infusion forms	0.0847	0.9338	0.1445	0.8853	0.8346
d) Corticosteroids in oral or infusion forms	0.3295	0.8330	0.2069	0.9038	0.7742
e) Treatment of tonsillitis	0.0046	1.0000	1.0000	0.8837	0.8838
**Combined diagnostic and conditions**					
2) Codes of repetitive hematuria or glomerulonephritis and					
6) Measurement of serum IgA at least twice	0.3638	0.9507	0.4938	0.9187	0.8822
and c) use of corticosteroids in infusion forms	0.0984	0.9940	0.6825	0.8929	0.8894
7) Needle biopsy with immunostaining/immunofluorescence	0.3135	0.9619	0.5209	0.9138	0.8862
and c) use of corticosteroids in infusion forms	0.1030	0.9943	0.7031	0.8934	0.8902
a) Specific coronary artery dilators (including dilazep)	0.4600	0.9020	0.3829	0.9267	0.8504
b) Renin–angiotensin inhibitors	0.0824	0.9797	0.3495	0.8898	0.8750
c) Corticosteroids in infusion forms	0.1121	0.9924	0.6622	0.8942	0.8897
d) Treatment of tonsillitis	0.0046	1.0000	1.0000	0.8837	0.8838
6) Two or more times of measurement of serum IgA and					
7) Needle biopsy with immunostaining/immunofluorescence	0.2700	0.9456	0.3960	0.9074	0.8667
and c) use of corticosteroids in oral or infusion forms	0.1785	0.9634	0.3920	0.8987	0.8718
a) Specific antiplatelets codes	0.2929	0.8938	0.2672	0.9053	0.8237
b) Renin–angiotensin system inhibitors	0.0343	0.9785	0.1744	0.8846	0.8683
c) Corticosteroids in infusion forms	0.1213	0.9658	0.3193	0.8926	0.8672

Sn, sensitivity; Sp: Specificity; PV+, positive predictive value; PV, negative predictive value; Ac: Accuracy.

Kidney biopsy with immunofluorescence was performed in 36.6% and 11.6% of patients with and without IgAN, respectively.

### Analysis of the test performance of manual criteria

As mentioned above, the specificity of the IgAN code was not the highest among the related variables. The three best PV+ are shown in the criteria “any N02x or N03x code combined with tonsil treatment,” “any N02x or N03x code combined with both needle biopsy with immunostaining/immunofluorescence and corticosteroid infusion,” and “code of IgAN.” The PV+ values for each criterion were 1, 0.7031, and 0.6825, respectively. The highest sensitivity (0.6316) was shown in the criterion of “measurement of serum IgA.” However, no single item achieved a coexistence of sensitivity and specificity >80%.

The AUC for manually created criteria was 0.715. Both sensitivity and specificity were below 0.8 at the cutoff set by Youden’s method. The ROC curve is shown in **S1 Fig**.

### Result of machine learning

**Fig 3:**
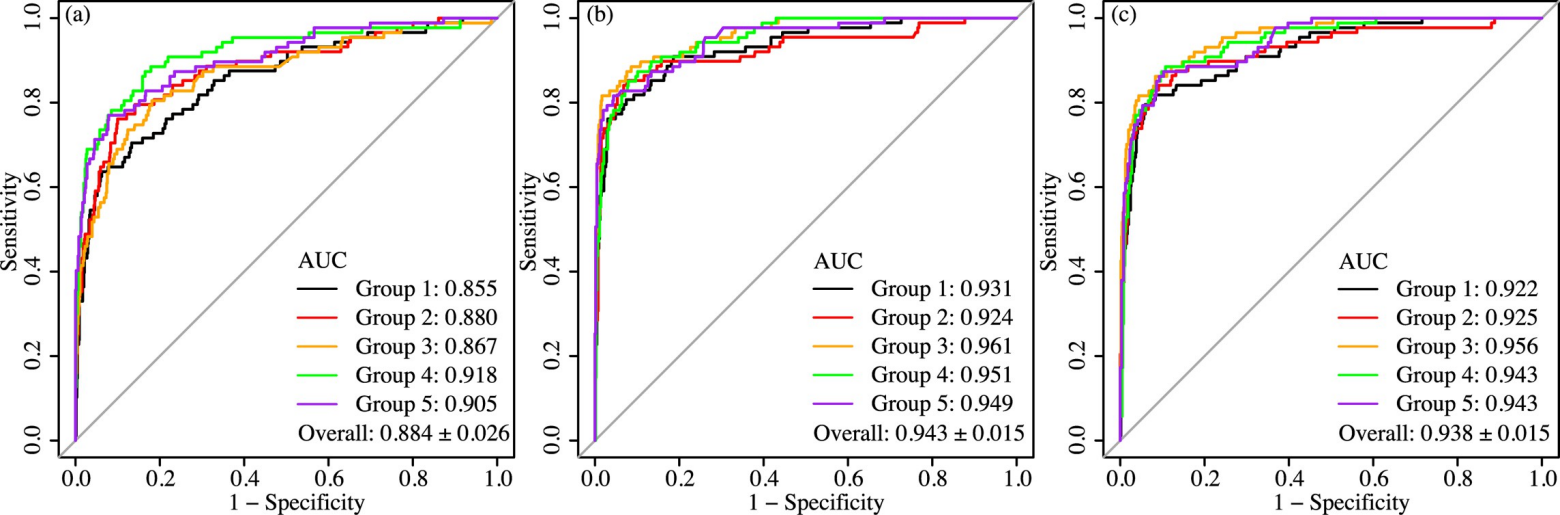
ROC curves using results of machine learning in patterns (a), (b), and (c). (a) The raw data; (b) Analyzed data with compiled codes of medication and diagnosis, except for diagnostic codes in nephrology; (c) Analyzed data with compiled codes of medication and all diagnoses. The group numbers in the legend indicate the group used in verification process (other four groups are used for training in XGboost).

**Fig 4:**
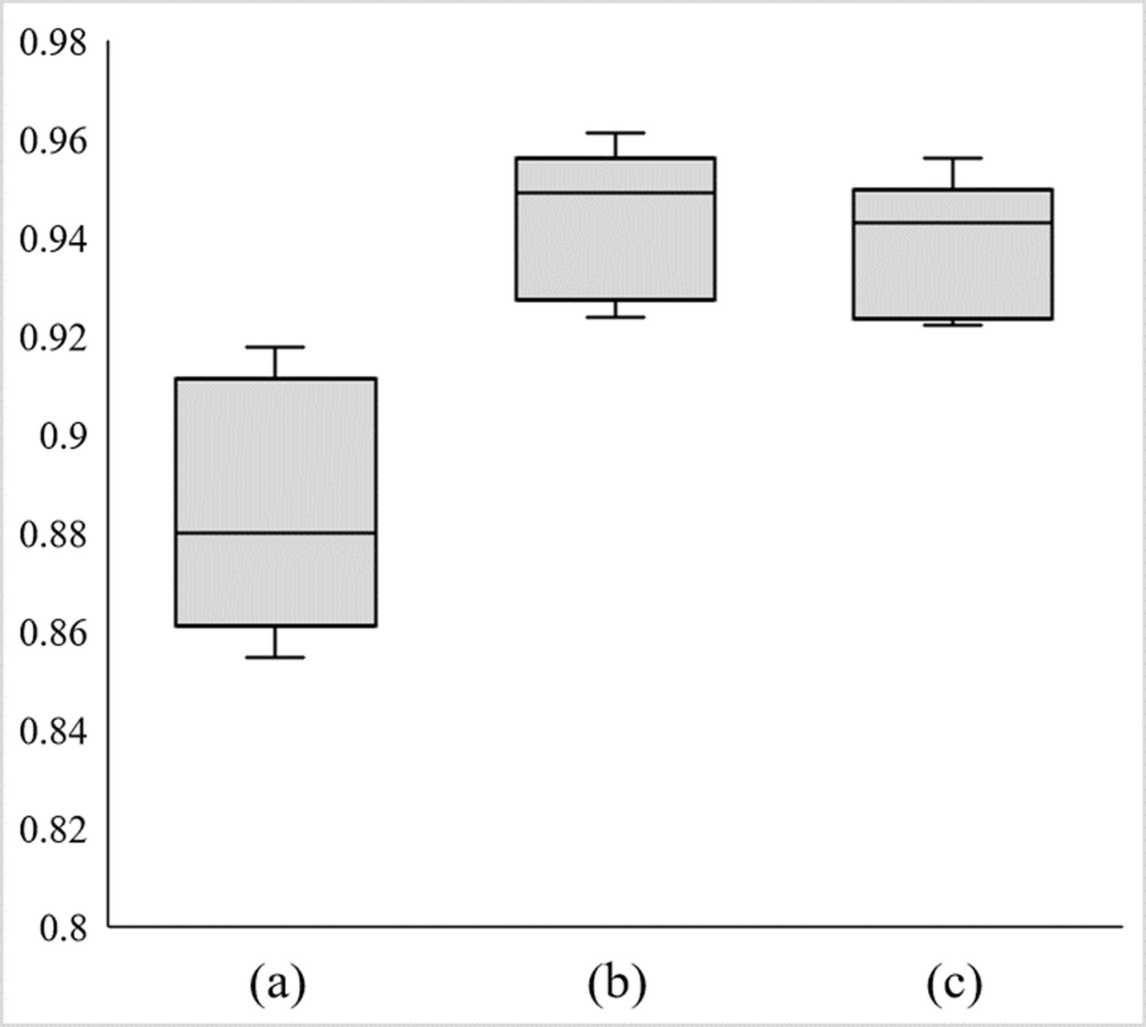
Boxplot of distribution of AUCs for five ROC curves in patterns (a), (b), and (c). (a) The raw data; (b) Data with compiled codes of medication and diagnosis, except for diagnostic codes in nephrology, were analyzed; (c) Data with compiled codes of medication and all diagnoses were analyzed.

**Fig 3**presents the results of plotting the ROC curve of the classifier created by machine learning. AUCs for patterns (a), (b), and (c) were 0.884 ± 0.026, 0.943 ± 0.015, and 0.937 ± 0.014, respectively (**Fig 4**). The process yielded better results in terms of the AUC in patterns (b) and (c), which used clinically utilized data, than in pattern (a) or manual analysis. The number of variables in pattern (b) (showing the highest average AUC) was 1836. The list of all items is shown in **S1 File** (in Japanese). The top 10 explanatory variables contributing to efficient classification for each cross-validation group are shown in **S2 Table**. The explanatory variables that appeared in the top ten in all patterns were age at the time of the oldest data, ICD-10 J3x (acute tonsillitis), ICD-10 N028 (IgAN), and ICD-10 N039 (chronic glomerulonephritis, undifferentiated).

## Discussion

We tried to identify true patients with IgAN from billing data in view of the inaccuracy of IgAN coding, and subsequently compared the results of manual analysis for the diagnostic performance of IgAN with the results obtained using machine learning. Although a high PV+ was observed in some manual criteria, the sensitivities were extremely low for practical use in all criteria. Considering the low prevalence of IgAN in the general population, a small number of patients were diagnosed using the manual criteria. Thus, machine learning using clinical datasets of medical bills showed far better performance than analysis using the manual method for diagnosing IgAN. We successfully improved the possibility of detecting patients with specific diseases by using medical billing data only. To the best of our knowledge, this is the first report on the use of machine-learning technology to extract true-diagnosed patients from medical billing data without data from biopsy reports, laboratory tests, or clinical records written by physicians.

The strength of these results is their high sensitivity and specificity. The specificities for diagnosing IgAN by machine learning using the utilized datasets surpassed 90% under the set conditions, which is not inferior to the diagnostic code of IgAN alone. Moreover, the sensitivity of machine learning using the utilized datasets was significantly better than that of the IgAN diagnostic code. We believe that this performance would suffice to create an artificial cohort of patients who are strongly predicted to have IgAN, with fewer omissions. It would be possible to establish a virtual cohort of patients that are strongly expected to be diagnosed with IgAN from the medical billing database using this method, which is not susceptible to overdiagnosis or diagnosis of diagnostic codes in the billing data. This technology enables the investigation of the long-term effectiveness of IgAN treatment. Moreover, this methodology can be applied to other diseases in the future.

This study had some limitations. First, the external validity was not guaranteed because the data were collected from a nephrology department at a single center. The direct use of a classifier in other departments or institutions has the potential to cause bias in the local practice of departments, hospitals, or regions. For example, the top 10 factors that were most powerful in distinguishing patients with IgAN from those without IgAN by machine learning, including antiplasmin drugs and carbazochrome sodium sulfonate, were components of the regular clinical pathway applied in our hospital. To solve this problem, billing data from other institutions should be used for the learning or testing processes.

The second limitation is the periodic revision of rules for creating bills. Many codes were added, deleted, or divided into subitems. It is almost impossible to capture all of these changes and reflect them in a single classifier. Thus, some factors used in the analysis may not have been available for different periods. Therefore, it is necessary to cautiously apply the classifier directly to data recorded from different periods. We believe that updating the classifiers using the data extracted during the update period could be a solution to this limitation.

## Conclusion

Compared with manual analysis or the application of machine learning to raw billing data, the application of machine-learning technology to well-utilized billing data showed high predictive performance for detecting patients with IgAN. This technology will help construct disease-specific cohorts from the billing data and may contribute to research on various diseases for which it is difficult to conduct a prospective interventional study in nephrology and many other clinical specialties.

## Supporting information

S1 Fig ROC curve plotted by manually constructed scoring based on the related diagnostic codes and treatment for IgAN. The point on the ROC shows the best cutoff point by Youden’s method (Sensitivity + specificity − 1) and the sensitivity and specificity at the cutoff.
(TIF)

S1 Table Representative hyperparameters for XGboost tuned by Optuna. “eta” defines the learning rate; “round” defines the round number of boosting; “max_depth” defines the maximum depth of the hierarchy of each decision tree; “colsample_bytree” is the probability that each variable is adopted as the source of each decision tree.
(DOCX)

S2 Table The top 10 explanatory variables in each group of pattern (b). F score shows the degrees of contribution of the variable within each group. The absolute values of this score are not comparable straddling different groups. The score does not mean the possibility of IgAN, but the degree of contribution for both inclusion and exclusion of IgAN.
(DOCX)

S1 File All components of variables in pattern (b) from extracted bills. (DOCX)
