# Supplementary figures and images for: Machine-learning-based identification of patients with IgA nephropathy using a computerized medical billing database
Source: PLoS One. 2024 Dec 5;19(12):e0312915. doi: 10.1371/journal.pone.0312915 (PMC11620576; doi:10.1371/journal.pone.0312915)

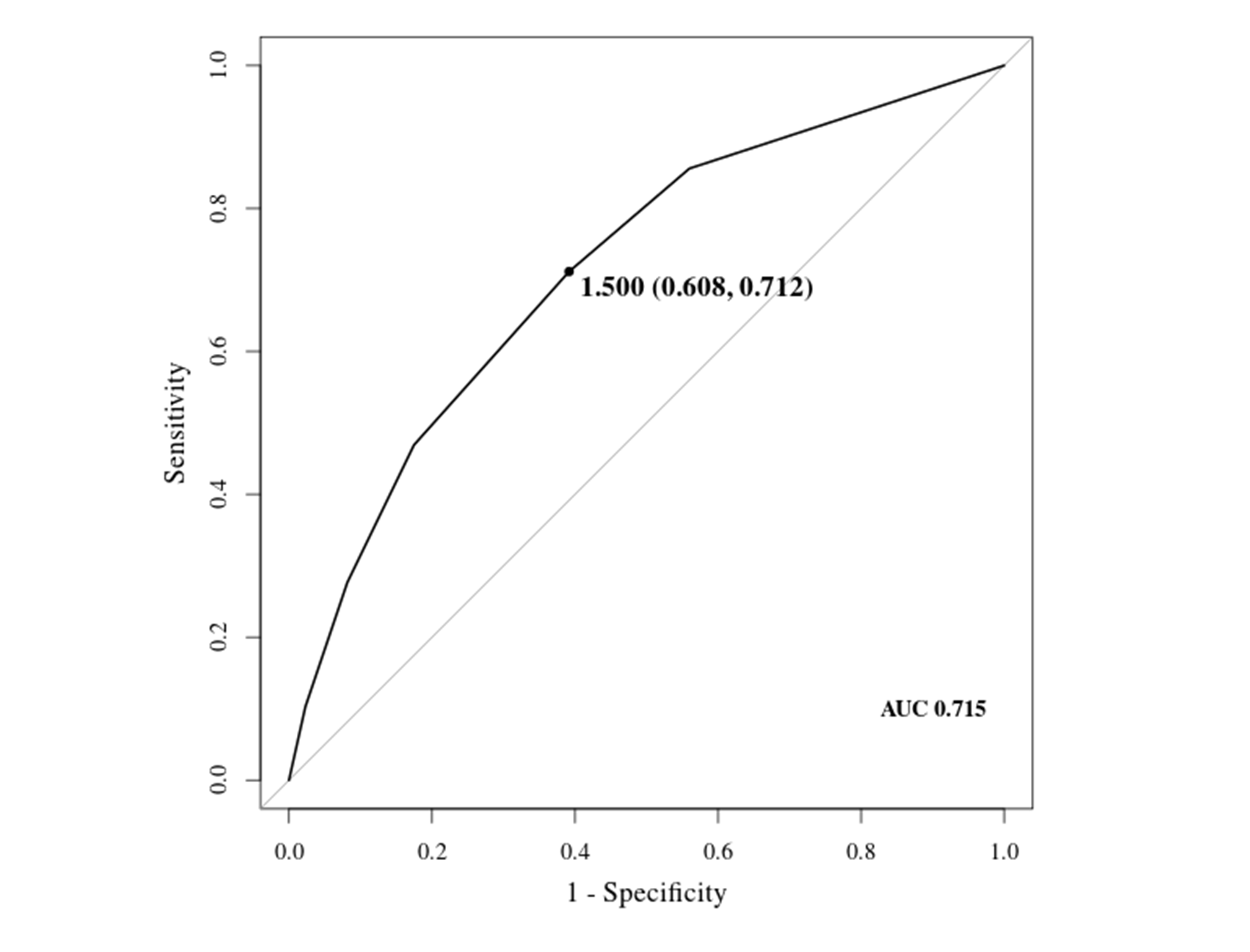

Supplement: S1 Fig — The point on the ROC shows the best cutoff point by Youden’s method (Sensitivity + specificity − 1) and the sensitivity and specificity at the cutoff. (TIF) [file pone.0312915.s001.TIF]
