# Supplementary material for: Machine-learning-based identification of patients with IgA nephropathy using a computerized medical billing database
Source: PLoS One. 2024 Dec 5;19(12):e0312915. doi: 10.1371/journal.pone.0312915 (PMC11620576; doi:10.1371/journal.pone.0312915)
Supplement: S1 Table — “eta” defines the learning rate; “round” defines the round number of boosting; “max_depth” defines the maximum depth of the hierarchy of each decision tree; “colsample_bytree” is the probability that each variable is adopted as the source of each decision tree. (DOCX) [file pone.0312915.s002.docx]

**Supplementary Table1**

|  | eta | round |  | max_depth | colsample_bytree |
| --- | --- | --- | --- | --- | --- |
| **Pattern (a)** |  |  |  |  |  |
| Group 1 | 0.05 | 193 |  | 6 | 0.379443855 |
| Group 2 | 0.05 | 254 |  | 6 | 0.256846276 |
| Group 3 | 0.05 | 46 |  | 6 | 0.583262556 |
| Group 4 | 0.05 | 174 |  | 6 | 0.392593486 |
| Group 5 | 0.05 | 294 |  | 5 | 0.478959414 |
| **Pattern (b)** |  |  |  |  |  |
| Group 1 | 0.05 | 108 |  | 6 | 0.326938747 |
| Group 2 | 0.05 | 54 |  | 4 | 0.430549797 |
| Group 3 | 0.05 | 92 |  | 6 | 0.446299163 |
| Group 4 | 0.05 | 87 |  | 6 | 0.420129564 |
| Group 5 | 0.05 | 93 |  | 3 | 0.982961416 |
| **Pattern (c)** |  |  |  |  |  |
| Group 1 | 0.05 | 53 |  | 6 | 0.507969186 |
| Group 2 | 0.05 | 199 |  | 5 | 0.201446041 |
| Group 3 | 0.05 | 199 |  | 3 | 0.284860558 |
| Group 4 | 0.05 | 73 |  | 5 | 0.602520856 |
| Group 5 | 0.05 | 53 |  | 4 | 0.594184115 |
