# Supplementary material for: Machine-learning-based identification of patients with IgA nephropathy using a computerized medical billing database
Source: PLoS One. 2024 Dec 5;19(12):e0312915. doi: 10.1371/journal.pone.0312915 (PMC11620576; doi:10.1371/journal.pone.0312915)
Supplement: S2 Table — F score shows the degrees of contribution of the variable within each group. The absolute values of this score are not comparable straddling different groups. The score does not mean the possibility of IgAN, but the degree of contribution for both inclusion and exclusion of IgAN. (DOCX) [file pone.0312915.s003.docx]

**Supplementary Table 2**

| **Group 1** | F Score |
| --- | --- |
| Age at the point of the oldest data | 77 |
| ICD-10 J3x (acute tonsilitis) | 37 |
| ICD-10 N028 (IgA nephropathy) | 35 |
| ICD-10 N039 (chronic glomerulonephritis, undifferentiated) | 33 |
| ICD-10 J0x (acute rhinopharyngitis) | 22 |
| ICD-10 K2x (dental caries) | 21 |
| ICD-10 E1x (diabetes mellitus) | 20 |
| YJ 21390x-21393x (other diuretics, oral) | 18 |
| YJ 33274x-33277x (antiplasmin drugs, infusion) | 16 |
| ICD-10 M3x (systemic connective tissue disorders) | 16 |
| **Group 2** |  |
| Age at the point of the oldest data | 29 |
| ICD-10 N028 (IgA nephropathy) | 27 |
| ICD-10 N039 (chronic glomerulonephritis, undifferentiated) | 24 |
| ICD-10 J3x (acute tonsilitis) | 19 |
| Preparation of the tissue specimen for electron microscopy | 14 |
| ICD-10 M3x (systemic connective tissue disorders) | 13 |
| YJ 33214x-33217x (carbazochrome, infusion) | 12 |
| ICD-10 N059 (Unspecified nephritic syndrome with unspecified morphologic changes) | 11 |
| ICD-10 M1x (Inflammatory polyarthropathies (in part) and osteoarthritis (in part)) | 11 |
| Preparation of the tissue specimen for immunostaining | 9 |
| **Group 3** |  |
| Age at the point of the oldest data | 71 |
| ICD-10 N028 (IgA nephropathy) | 40 |
| ICD-10 N039 (chronic glomerulonephritis, undifferentiated) | 35 |
| ICD-10 J3x (acute tonsilitis) | 34 |
| Laboratory test: Urine N-acetylglucosaminidase | 22 |
| Ultrasonography (tomography, chest or abdomen) | 19 |
| ICD-10 K2x (dental caries) | 15 |
| ICD-10 N052 (Unspecified nephritic syndrome, diffuse membranous glomerulonephritis) | 15 |
| Laboratory test: Antinuclear antibody (other than immunofluorescence) | 14 |
| YJ 33274x-33277x (antiplasmin drugs, infusion) | 13 |
| **Group 4** |  |
| Age at the point of the oldest data | 110 |
| ICD-10 N028 (IgA nephropathy) | 35 |
| ICD-10 N052 (Unspecified nephritic syndrome, diffuse membranous glomerulonephritis) | 32 |
| ICD-10 N039 (chronic glomerulonephritis, undifferentiated) | 31 |
| ICD-10 J3x (acute tonsilitis) | 27 |
| YJ 2149x (angiotensin II receptor blockers or direct renin inhibitors) | 22 |
| ICD-10 M3x (systemic connective tissue disorders) | 22 |
| Laboratory test: Antinuclear antibody (other than immunofluorescence) | 22 |
| ICD-10 E1x (diabetes mellitus) | 21 |
| ICD-10 I1x (hypertension) | 18 |
| **Group 5** |  |
| ICD-10 N028 (IgA nephropathy) | 68 |
| ICD-10 N039 (chronic glomerulonephritis, undifferentiated) | 36 |
| Age at the point of the oldest data | 25 |
| ICD-10 J3x (acute tonsilitis) | 24 |
| YJ 21390x-21393x (other diuretics, oral) | 24 |
| Laboratory test: HDL-cholesterol | 20 |
| Preparation of the tissue specimen for electron microscopy | 19 |
| ICD-10 M1x (Inflammatory polyarthropathies (in part) and osteoarthritis (in part)) | 17 |
| Laboratory test: Immunoelectrophoresis | 13 |
| YJ 33214x-33217x (carbazochrome, infusion) | 13 |
