# Supplementary material for: Machine-learning-based identification of patients with IgA nephropathy using a computerized medical billing database
Source: PLoS One. 2024 Dec 5;19(12):e0312915. doi: 10.1371/journal.pone.0312915 (PMC11620576; doi:10.1371/journal.pone.0312915)
Supplement: S1 File — (DOCX) [file pone.0312915.s004.docx]

**Supplementary materials**

**Supplementary material 1:** All components of variables in pattern (b) from extracted bills:

入外区分,性別,年齢（初記録時）,診療科コード,科名,入院中他科コード,入院中他科名,受診科コード,受診科名,ICD10_A0,ICD10_A1,ICD10_A3,ICD10_A4,ICD10_A5,ICD10_A6,ICD10_A8,ICD10_B0,ICD10_B1,ICD10_B2,ICD10_B3,ICD10_B4,ICD10_B5,ICD10_B9,ICD10_C0,ICD10_C1,ICD10_C2,ICD10_C3,ICD10_C4,ICD10_C5,ICD10_C6,ICD10_C7,ICD10_C8,ICD10_C9,ICD10_D0,ICD10_D1,ICD10_D2,ICD10_D3,ICD10_D4,ICD10_D5,ICD10_D6,ICD10_D7,ICD10_D8,ICD10_E0,ICD10_E1,ICD10_E2,ICD10_E4,ICD10_E5,ICD10_E6,ICD10_E7,ICD10_E8,ICD10_F0,ICD10_F1,ICD10_F2,ICD10_F3,ICD10_F4,ICD10_F5,ICD10_F7,ICD10_F8,ICD10_F9,ICD10_G0,ICD10_G1,ICD10_G2,ICD10_G3,ICD10_G4,ICD10_G5,ICD10_G6,ICD10_G7,ICD10_G8,ICD10_G9,ICD10_H0,ICD10_H1,ICD10_H2,ICD10_H3,ICD10_H4,ICD10_H5,ICD10_H6,ICD10_H7,ICD10_H8,ICD10_H9,ICD10_I0,ICD10_I1,ICD10_I2,ICD10_I3,ICD10_I4,ICD10_I5,ICD10_I6,ICD10_I7,ICD10_I8,ICD10_I9,ICD10_J0,ICD10_J1,ICD10_J2,ICD10_J3,ICD10_J4,ICD10_J6,ICD10_J7,ICD10_J8,ICD10_J9,ICD10_K0,ICD10_K1,ICD10_K2,ICD10_K3,ICD10_K4,ICD10_K5,ICD10_K6,ICD10_K7,ICD10_K8,ICD10_K9,ICD10_L0,ICD10_L1,ICD10_L2,ICD10_L3,ICD10_L4,ICD10_L5,ICD10_L6,ICD10_L7,ICD10_L8,ICD10_L9,ICD10_M0,ICD10_M1,ICD10_M2,ICD10_M3,ICD10_M4,ICD10_M5,ICD10_M6,ICD10_M7,ICD10_M8,ICD10_M9,ICD10_N009_急性腎炎症候群: 詳細不明,ICD10_N014_急速進行性腎炎症候群: びまん性管内増殖性糸球体腎炎,ICD10_N017_急速進行性腎炎症候群: びまん性半月体形成性糸球体腎炎,ICD10_N019_急速進行性腎炎症候群: 詳細不明,ICD10_N028_反復性及び持続性血尿: その他,ICD10_N029_反復性及び持続性血尿: 詳細不明,ICD10_N030_慢性腎炎症候群: 軽微糸球体変化,ICD10_N032_慢性腎炎症候群: びまん性膜性糸球体腎炎,ICD10_N033_慢性腎炎症候群: びまん性メザンギウム増殖性糸球体腎炎,ICD10_N039_慢性腎炎症候群: 詳細不明,ICD10_N040_ネフローゼ症候群: 軽微糸球体変化,ICD10_N041_ネフローゼ症候群: 巣状及び分節状糸球体変化,ICD10_N042_ネフローゼ症候群: びまん性膜性糸球体腎炎,ICD10_N044_ネフローゼ症候群: びまん性管内増殖性糸球体腎炎,ICD10_N046_ネフローゼ症候群: デンスデポジット病,ICD10_N048_ネフローゼ症候群: その他,ICD10_N049_ネフローゼ症候群: 詳細不明,ICD10_N051_詳細不明の腎炎症候群: 巣状及び分節状糸球体変化,ICD10_N052_詳細不明の腎炎症候群: びまん性膜性糸球体腎炎,ICD10_N053_詳細不明の腎炎症候群: びまん性メザンギウム増殖性糸球体腎炎,ICD10_N054_詳細不明の腎炎症候群: びまん性管内増殖性糸球体腎炎,ICD10_N055_詳細不明の腎炎症候群: びまん性メザンギウム毛細管性糸球体腎炎,ICD10_N056_詳細不明の腎炎症候群: デンスデポジット病,ICD10_N057_詳細不明の腎炎症候群: びまん性半月体形成性糸球体腎炎,ICD10_N058_詳細不明の腎炎症候群: その他,ICD10_N059_詳細不明の腎炎症候群: 詳細不明,"ICD10_N079_遺伝性腎症, 他に分類されないもの: 詳細不明",ICD10_N10_急性尿細管間質性腎炎,ICD10_N110_非閉塞性逆流性慢性腎盂腎炎,"ICD10_N119_慢性尿細管間質性腎炎, 詳細不明","ICD10_N12_尿細管間質性腎炎, 急性又は慢性と明示されないもの","ICD10_N131_尿管狭窄を伴う水腎症, 他に分類されないもの",ICD10_N132_腎結石性及び尿管結石性閉塞を伴う水腎症,ICD10_N133_その他及び詳細不明の水腎症,ICD10_N134_水尿管,ICD10_N135_水腎症を伴わない尿管の屈曲及び狭窄,ICD10_N136_膿腎,ICD10_N137_膀胱尿管逆流性尿路疾患,"ICD10_N142_詳細不明の薬物, 薬剤又は生物学的製剤により誘発された腎症",ICD10_N151_腎膿瘍及び腎周囲膿瘍,"ICD10_N159_腎尿細管間質性疾患, 詳細不明",ICD10_N170_尿細管壊死を伴う急性腎不全,ICD10_N178_その他の急性腎不全,"ICD10_N179_急性腎不全, 詳細不明",ICD10_N180_Deleted,"ICD10_N182_慢性腎不全, 2期","ICD10_N183_慢性腎不全, 3期","ICD10_N184_慢性腎不全, 4期","ICD10_N185_慢性腎不全, 5期",ICD10_N188_Deleted,"ICD10_N189_慢性腎不全, 詳細不明",ICD10_N19_詳細不明の腎不全,ICD10_N200_腎結石,ICD10_N201_尿管結石,ICD10_N202_尿管結石を伴う腎結石,"ICD10_N209_尿路結石, 詳細不明",ICD10_N211_尿道結石,"ICD10_N219_下部尿路結石, 詳細不明",ICD10_N250_腎性骨異栄養症,ICD10_N251_腎性尿崩症,ICD10_N258_腎尿細管機能障害から生じたその他の障害,"ICD10_N259_腎尿細管機能障害から生じた障害, 詳細不明",ICD10_N26_詳細不明の萎縮腎,ICD10_N280_腎虚血及び腎梗塞,ICD10_N281_腎嚢胞,ICD10_N288_腎及び尿管のその他の明示された障害,"ICD10_N289_腎及び尿管の障害, 詳細不明",ICD10_N3,ICD10_N4,ICD10_N6,ICD10_N7,ICD10_N8,ICD10_N9,ICD10_O0,ICD10_O1,ICD10_O2,ICD10_O3,ICD10_O9,ICD10_P7,ICD10_Q0,ICD10_Q1,ICD10_Q2,ICD10_Q3,ICD10_Q4,ICD10_Q5,ICD10_Q6,ICD10_Q7,ICD10_Q8,ICD10_Q9,ICD10_R0,ICD10_R1,ICD10_R2,ICD10_R3,ICD10_R4,ICD10_R5,ICD10_R6,ICD10_R7,ICD10_R8,ICD10_R9,ICD10_S0,ICD10_S1,ICD10_S2,ICD10_S3,ICD10_S4,ICD10_S5,ICD10_S6,ICD10_S7,ICD10_S8,ICD10_S9,ICD10_T0,ICD10_T1,ICD10_T4,ICD10_T5,ICD10_T6,ICD10_T7,ICD10_T8,ICD10_T9,ICD10_U0,ICD10_Z0,ICD10_Z1,ICD10_Z2,ICD10_Z3,ICD10_Z4,ICD10_Z5,ICD10_Z8,ICD10_Z9,ICD10_nan,ICD10_T2,ICD10_O6,ICD10_E3,ICD10_O8,ICD10_P0,ICD10_N5,YJ_1115_溶性バルビツール酸系及び溶性チオバルビツール酸系製剤_注射,YJ_1116_亜酸化窒素製剤_外用,YJ_1119_その他の全身麻酔剤_外用,YJ_1119_その他の全身麻酔剤_注射,YJ_1124_ベンゾジアゼピン系製剤_内服,YJ_1124_ベンゾジアゼピン系製剤_外用,YJ_1124_ベンゾジアゼピン系製剤_注射,YJ_1125_バルビツール酸系及びチオバルビツール酸系製剤_内服,YJ_1129_その他の催眠鎮静剤、抗不安剤_内服,YJ_1129_その他の催眠鎮静剤、抗不安剤_注射,YJ_1132_ヒダントイン系製剤_内服,YJ_1132_ヒダントイン系製剤_注射,YJ_1139_その他の抗てんかん剤_内服,YJ_1139_その他の抗てんかん剤_注射,YJ_1141_アニリン系製剤；メフェナム酸、フルフェナム酸等_内服,YJ_1141_アニリン系製剤；メフェナム酸、フルフェナム酸等_外用,YJ_1141_アニリン系製剤；メフェナム酸、フルフェナム酸等_注射,YJ_1143_サリチル酸系製剤；アスピリン等_内服,YJ_1145_インドメタシン製剤_内服,YJ_1147_フェニル酢酸系製剤_内服,YJ_1147_フェニル酢酸系製剤_外用,YJ_1148_塩基性消炎鎮痛剤_内服,YJ_1149_その他の解熱鎮痛消炎剤_内服,YJ_1149_その他の解熱鎮痛消炎剤_外用,YJ_1149_その他の解熱鎮痛消炎剤_注射,YJ_1161_アマンタジン製剤_内服,YJ_1162_ビペリデン製剤_内服,YJ_1169_その他の抗パーキンソン剤_内服,YJ_1169_その他の抗パーキンソン剤_外用,YJ_1171_クロルプロマジン製剤_内服,YJ_1171_クロルプロマジン製剤_注射,YJ_1172_フェノチアジン系製剤_内服,YJ_1174_イミプラミン系製剤_内服,YJ_1179_その他の精神神経用剤_内服,YJ_1179_その他の精神神経用剤_注射,YJ_1180_総合感冒剤_内服,YJ_1190_その他の中枢神経系用薬_内服,YJ_1190_その他の中枢神経系用薬_外用,YJ_1190_その他の中枢神経系用薬_注射,YJ_1214_キシリジン系製剤_内服,YJ_1214_キシリジン系製剤_外用,YJ_1214_キシリジン系製剤_注射,YJ_1219_その他の局所麻酔剤_内服,YJ_1219_その他の局所麻酔剤_外用,YJ_1224_コリン系製剤；コハク酸コリン等_注射,YJ_1229_その他の骨格筋弛緩剤_内服,YJ_1229_その他の骨格筋弛緩剤_注射,YJ_1231_四級アンモニウム塩製剤；メタンテリンブロミド等_内服,YJ_1233_ネオスチグミン系製剤_注射,YJ_1239_その他の自律神経剤_内服,YJ_1242_アトロピン系製剤_内服,YJ_1242_アトロピン系製剤_注射,YJ_1243_パパベリン系製剤_注射,YJ_1244_マグネシウム塩製剤；硫酸マグネシウム注射液等_注射,YJ_1249_その他の鎮痙剤_内服,YJ_1311_散瞳剤；ホマトロピン等_外用,YJ_1312_縮瞳剤；ピロカルピン等_外用,YJ_1315_眼科用コルチゾン製剤；コルチゾン点眼液及び眼軟膏剤_外用,YJ_1319_その他の眼科用剤_外用,YJ_1324_耳鼻科用血管収縮剤_外用,YJ_1325_耳鼻科用抗生物質製剤_外用,YJ_1329_その他の耳鼻科用剤_外用,YJ_1339_その他の鎮暈剤_内服,YJ_2113_ジギタリス製剤_内服,YJ_2113_ジギタリス製剤_注射,YJ_2115_カフェイン系製剤_注射,YJ_2119_その他の強心剤_内服,YJ_2119_その他の強心剤_注射,YJ_2123_β−遮断剤_内服,YJ_2123_β−遮断剤_注射,YJ_2129_その他の不整脈用剤_内服,YJ_2129_その他の不整脈用剤_注射,YJ_2132_チアジド系製剤_内服,YJ_2133_抗アルドステロン製剤；トリアムテレン等_内服,YJ_2133_抗アルドステロン製剤；トリアムテレン等_注射,YJ_2134_炭酸脱水酵素阻害剤_内服,YJ_2135_クロルベンゼンスルホンアミド系製剤_内服,YJ_2139_その他の利尿剤_内服,YJ_2139_その他の利尿剤_注射,YJ_2142_ヒドララジン製剤_内服,YJ_2144_アンジオテンシン変換酵素阻害剤_内服,YJ_2145_メチルドパ製剤_内服,YJ_2149_その他の血圧降下剤_内服,YJ_2149_その他の血圧降下剤_外用,YJ_2149_その他の血圧降下剤_注射,YJ_2160_血管収縮剤_内服,YJ_2160_血管収縮剤_注射,YJ_2171_冠血管拡張剤_内服,YJ_2171_冠血管拡張剤_外用,YJ_2171_冠血管拡張剤_注射,YJ_2179_その他の血管拡張剤_注射,YJ_2183_クロフィブラート系製剤_内服,YJ_2189_その他の高脂血症用剤_内服,YJ_2190_その他の循環器官用薬_内服,YJ_2190_その他の循環器官用薬_注射,YJ_2219_その他の呼吸促進剤_注射,YJ_2221_エフェドリン及びマオウ製剤_注射,YJ_2223_デキストロメトルファン製剤_内服,YJ_2229_その他の鎮咳剤_内服,YJ_2233_システイン系製剤_内服,YJ_2234_ブロムヘキシン製剤_内服,YJ_2234_ブロムヘキシン製剤_外用,YJ_2234_ブロムヘキシン製剤_注射,YJ_2239_その他の去痰剤_内服,YJ_2242_コデイン系製剤（家庭麻薬）_内服,YJ_2249_その他の鎮咳去痰剤_内服,YJ_2251_キサンチン系製剤_内服,YJ_2254_サルブタモール製剤_外用,YJ_2259_その他の気管支拡張剤_内服,YJ_2259_その他の気管支拡張剤_外用,YJ_2260_含嗽剤_外用,YJ_2290_その他の呼吸器官用薬_外用,YJ_2312_タンニン酸系製剤；タンニン酸アルブミン等_内服,YJ_2316_活性生菌製剤_内服,YJ_2318_ジメチコン製剤_内服,YJ_2319_その他の止瀉剤、整腸剤_内服,YJ_2325_Ｈ２遮断剤_内服,YJ_2325_Ｈ２遮断剤_注射,YJ_2329_その他の消化性潰瘍用剤_内服,YJ_2329_その他の消化性潰瘍用剤_注射,YJ_2331_消化酵素製剤_内服,YJ_2339_その他の健胃消化剤_内服,YJ_2344_無機塩製剤；炭酸水素ナトリウム等_内服,YJ_2349_その他の制酸剤_内服,YJ_2354_植物性製剤；センナ等_内服,YJ_2357_グリセリン製剤_外用,YJ_2359_その他の下剤、浣腸剤_内服,YJ_2359_その他の下剤、浣腸剤_外用,YJ_2362_胆汁酸製剤_内服,YJ_2391_鎮吐剤_内服,YJ_2391_鎮吐剤_注射,YJ_2399_他に分類されない消化器官用薬_内服,YJ_2399_他に分類されない消化器官用薬_外用,YJ_2399_他に分類されない消化器官用薬_注射,YJ_2411_ＡＣＴＨ製剤_注射,YJ_2414_脳下垂体後葉ホルモン製剤；オキシトシン等_注射,YJ_2419_その他の脳下垂体ホルモン剤_内服,YJ_2419_その他の脳下垂体ホルモン剤_外用,YJ_2431_甲状腺ホルモン製剤_内服,YJ_2432_抗甲状腺ホルモン製剤_内服,YJ_2439_その他の甲状腺、副甲状腺ホルモン剤_注射,YJ_2449_その他のたん白同化ステロイド剤_内服,YJ_2451_エピネフリン製剤_注射,YJ_2452_コルチゾン系製剤_内服,YJ_2452_コルチゾン系製剤_注射,YJ_2454_フッ素付加副腎皮質ホルモン製剤_内服,YJ_2454_フッ素付加副腎皮質ホルモン製剤_注射,YJ_2456_プレドニゾロン系製剤_内服,YJ_2456_プレドニゾロン系製剤_注射,YJ_2459_その他の副腎ホルモン剤_内服,YJ_2473_エストラジオール系製剤_外用,YJ_2475_エストリオール系製剤_内服,YJ_2491_循環ホルモン剤_内服,YJ_2492_すい臓ホルモン剤_注射,YJ_2499_他に分類されないホルモン剤（抗ホルモン剤を含む）_内服,YJ_2499_他に分類されないホルモン剤（抗ホルモン剤を含む）_注射,YJ_2529_その他の生殖器官用剤（性病予防剤を含む）_外用,YJ_2531_バッカク類製剤_注射,YJ_2559_その他の痔疾用剤_内服,YJ_2559_その他の痔疾用剤_外用,YJ_2590_その他の泌尿生殖器官及び肛門用薬_内服,YJ_2612_ヨウ素化合物；ヨードチンキ等_外用,YJ_2616_石鹸類製剤_外用,YJ_2619_その他の外皮用殺菌消毒剤_外用,YJ_2633_外用サルファ製剤_外用,YJ_2634_外用抗生物質製剤；ペニシリン軟膏等_外用,YJ_2639_その他の化膿性疾患用剤_外用,YJ_2642_外用抗ヒスタミン製剤_外用,YJ_2646_副腎皮質ホルモン製剤_外用,YJ_2647_抗生物質及び副腎皮質ホルモン混合製剤_外用,YJ_2649_その他の鎮痛、鎮痒、収斂、消炎剤_外用,YJ_2652_外用サリチル酸系製剤_外用,YJ_2655_イミダゾール系製剤_外用,YJ_2659_その他の寄生性皮膚疾患用剤_外用,YJ_2669_その他の皮膚軟化剤（腐食剤を含む）_外用,YJ_2691_外用ビタミン製剤；ビタミン軟膏等_外用,YJ_2699_他に分類されない外皮用薬_内服,YJ_2699_他に分類されない外皮用薬_外用,YJ_3112_合成ビタミンＤ製剤_内服,YJ_3112_合成ビタミンＤ製剤_注射,YJ_3122_ビタミンＢ１誘導体製剤_内服,YJ_3131_ビタミンＢ２剤_内服,YJ_3133_パントテン酸系製剤_注射,YJ_3134_ビタミンＢ６剤_内服,YJ_3135_葉酸製剤_内服,YJ_3136_ビタミンＢ１２剤_内服,YJ_3136_ビタミンＢ１２剤_注射,YJ_3140_ビタミンＣ剤_内服,YJ_3140_ビタミンＣ剤_注射,YJ_3150_ビタミンＥ剤_内服,YJ_3160_ビタミンＫ剤_内服,YJ_3160_ビタミンＫ剤_注射,YJ_3179_その他の混合ビタミン剤（ビタミンＡ・Ｄ混合製剤を除く）_内服,YJ_3179_その他の混合ビタミン剤（ビタミンＡ・Ｄ混合製剤を除く）_注射,YJ_3211_乳酸カルシウム製剤_内服,YJ_3213_グルコン酸カルシウム製剤_注射,YJ_3215_ハロゲン化カルシウム製剤；塩化カルシウム、臭化カルシウム等_注射,YJ_3219_その他のカルシウム剤_内服,YJ_3222_鉄化合物製剤（有機酸鉄を含む）_内服,YJ_3222_鉄化合物製剤（有機酸鉄を含む）_注射,YJ_3229_その他の無機質製剤_内服,YJ_3229_その他の無機質製剤_注射,YJ_3231_ブドウ糖製剤_内服,YJ_3231_ブドウ糖製剤_注射,YJ_3239_その他の糖類剤_注射,YJ_3253_混合アミノ酸製剤_内服,YJ_3253_混合アミノ酸製剤_注射,YJ_3259_その他のたん白アミノ酸製剤_内服,YJ_3259_その他のたん白アミノ酸製剤_注射,YJ_3299_他に分類されない滋養強壮薬_注射,YJ_3311_生理食塩液類_注射,YJ_3319_その他の血液代用剤_注射,YJ_3321_カルバゾクロム系製剤_内服,YJ_3321_カルバゾクロム系製剤_注射,YJ_3323_臓器性止血製剤_外用,YJ_3325_セルロース系製剤（可吸収性充填止血ガーゼ類を含む）_外用,YJ_3327_抗プラスミン剤_内服,YJ_3327_抗プラスミン剤_注射,YJ_3329_その他の止血剤_注射,YJ_3332_ジクマロール系製剤_内服,YJ_3334_ヘパリン製剤_注射,YJ_3339_その他の血液凝固阻止剤_内服,YJ_3339_その他の血液凝固阻止剤_外用,YJ_3339_その他の血液凝固阻止剤_注射,YJ_3399_他に分類されない血液・体液用薬_内服,YJ_3399_他に分類されない血液・体液用薬_外用,YJ_3399_他に分類されない血液・体液用薬_注射,YJ_3410_人工腎臓透析用剤_注射,YJ_3420_腹膜透析用剤_注射,YJ_3919_その他の肝臓疾患用剤_内服,YJ_3919_その他の肝臓疾患用剤_注射,YJ_3922_グルタチオン製剤_内服,YJ_3925_チオ硫酸ナトリウム製剤_注射,YJ_3929_その他の解毒剤_内服,YJ_3929_その他の解毒剤_注射,YJ_3941_コルヒチン製剤_内服,YJ_3942_プロベネシド製剤_内服,YJ_3943_アロプリノール製剤_内服,YJ_3949_その他の痛風治療剤_内服,YJ_3954_ウロキナーゼ製剤_注射,YJ_3959_その他の酵素製剤_注射,YJ_3961_スルフォニル尿素系製剤_内服,YJ_3962_ビグアナイド系製剤_内服,YJ_3969_その他の糖尿病用剤_内服,YJ_3992_アデノシン製剤_内服,YJ_3992_アデノシン製剤_注射,YJ_3999_他に分類されないその他の代謝性医薬品_内服,YJ_3999_他に分類されないその他の代謝性医薬品_注射,YJ_4211_クロルエチルアミン系製剤_内服,YJ_4211_クロルエチルアミン系製剤_注射,YJ_4219_その他のアルキル化剤_内服,YJ_4222_メトトレキサート製剤_内服,YJ_4224_シトシン系製剤_注射,YJ_4229_その他の代謝拮抗剤_内服,YJ_4235_アントラサイクリン系抗生物質製剤_注射,YJ_4240_抗腫瘍性植物成分製剤_注射,YJ_4291_その他の抗悪性腫瘍用剤_内服,YJ_4291_その他の抗悪性腫瘍用剤_注射,YJ_4299_他に分類されない腫瘍用薬_注射,YJ_4300_放射性医薬品_内服,YJ_4300_放射性医薬品_注射,YJ_4413_フェノチアジン系製剤_内服,YJ_4419_その他の抗ヒスタミン剤_内服,YJ_4419_その他の抗ヒスタミン剤_注射,YJ_4420_刺激療法剤_内服,YJ_4490_その他のアレルギー用薬_内服,YJ_5200_漢方製剤_内服,YJ_5900_その他の生薬及び漢方処方に基づく医薬品_内服,YJ_6112_リンコマイシン系抗生物質製剤_内服,YJ_6112_リンコマイシン系抗生物質製剤_注射,YJ_6113_バンコマイシン製剤_内服,YJ_6113_バンコマイシン製剤_注射,YJ_6119_その他の主としてグラム陽性菌に作用するもの_外用,YJ_6119_その他の主としてグラム陽性菌に作用するもの_注射,YJ_6122_モノバクタム系抗生物質製剤_注射,YJ_6123_アミノ糖系抗生物質製剤_内服,YJ_6123_アミノ糖系抗生物質製剤_注射,YJ_6131_ペニシリン系抗生物質製剤；合成ペニシリン_内服,YJ_6131_ペニシリン系抗生物質製剤；合成ペニシリン_注射,YJ_6132_セフェム系抗生物質製剤_内服,YJ_6132_セフェム系抗生物質製剤_注射,YJ_6133_オキサセフェム系抗生物質製剤_注射,YJ_6134_アミノ糖系抗生物質製剤_注射,YJ_6135_ホスホマイシン製剤_内服,YJ_6135_ホスホマイシン製剤_注射,YJ_6139_その他の主としてグラム陽性・陰性菌に作用するもの_内服,YJ_6139_その他の主としてグラム陽性・陰性菌に作用するもの_注射,YJ_6141_エリスロマイシン製剤_内服,YJ_6149_その他の主としてグラム陽性菌、マイコプラズマに作用するもの_内服,YJ_6149_その他の主としてグラム陽性菌、マイコプラズマに作用するもの_注射,YJ_6152_テトラサイクリン系抗生物質製剤_内服,YJ_6152_テトラサイクリン系抗生物質製剤_注射,YJ_6161_ストレプトマイシン系抗生物質製剤_注射,YJ_6164_リファンピシン製剤_内服,YJ_6173_アムホテリシンＢ製剤_内服,YJ_6173_アムホテリシンＢ製剤_注射,YJ_6179_その他の主としてカビに作用するもの_内服,YJ_6179_その他の主としてカビに作用するもの_注射,YJ_6199_他に分類されない抗生物質製剤_内服,YJ_6219_その他のサルファ剤_内服,YJ_6222_イソニアジド系製剤_内服,YJ_6223_ピラジナミド製剤_内服,YJ_6225_エタンブトール製剤_内服,YJ_6241_ピリドンカルボン酸系製剤_内服,YJ_6241_ピリドンカルボン酸系製剤_注射,YJ_6249_その他の合成抗菌剤_注射,YJ_6250_抗ウイルス剤_内服,YJ_6250_抗ウイルス剤_外用,YJ_6250_抗ウイルス剤_注射,YJ_6290_その他の化学療法剤_内服,YJ_6290_その他の化学療法剤_外用,YJ_6290_その他の化学療法剤_注射,YJ_6311_細菌ワクチン類_注射,YJ_6313_ウイルスワクチン類_注射,YJ_6322_トキソイド類_注射,YJ_6342_血液成分製剤_注射,YJ_6343_血漿分画製剤_注射,YJ_6393_精製ツベルクリン_注射,YJ_6399_他に分類されない生物学的製剤_注射,YJ_6419_その他の抗原虫剤_内服,YJ_6419_その他の抗原虫剤_注射,YJ_7121_油脂性基剤；ワセリン、パラフィン等_外用,YJ_7131_精製水類_内服,YJ_7131_精製水類_外用,YJ_7131_精製水類_注射,YJ_7142_シロップ製剤；単シロップ等_内服,YJ_7190_その他の調剤用薬_外用,YJ_7211_ヨウ素化合物製剤_内服,YJ_7211_ヨウ素化合物製剤_注射,YJ_7212_バリウム塩製剤_内服,YJ_7213_造影補助剤_内服,YJ_7214_配合製剤_注射,YJ_7219_その他のＸ線造影剤_注射,YJ_7223_内分泌機能検査用試薬_注射,YJ_7224_肝機能検査用試薬_注射,YJ_7225_腎機能検査用試薬_注射,YJ_7229_その他の機能検査用試薬_注射,YJ_7290_その他の診断用薬（体外診断用医薬品を除く）_内服,YJ_7290_その他の診断用薬（体外診断用医薬品を除く）_注射,YJ_7990_他に分類されない治療を主目的としない医薬品_内服,YJ_7990_他に分類されない治療を主目的としない医薬品_外用,YJ_7990_他に分類されない治療を主目的としない医薬品_注射,YJ_8114_モルヒネ系製剤_注射,YJ_8119_その他のあへんアルカロイド系麻薬_内服,YJ_8119_その他のあへんアルカロイド系麻薬_注射,YJ_8219_その他の合成麻薬_外用,YJ_8219_その他の合成麻薬_注射,Densan_code_ 0,Densan_code_000000000000,初診料,時間外加算（初診）,休日加算（初診）,深夜加算（初診）,時間外特例医療機関加算（初診）,初診料（同一日複数科受診時の２科目）,時間外加算（再診）（入院外）,休日加算（再診）（入院外）,深夜加算（再診）（入院外）,時間外特例医療機関加算（再診）（入院外）,外来診療料,同日外来診療料,外来診療料（同一日複数科受診時の２科目）,時間外加算（再診）（入院）,休日加算（再診）（入院）,深夜加算（再診）（入院）,時間外特例医療機関加算（再診）（入院）,妊婦加算（再診）,特定薬剤治療管理料１,特定薬剤治療管理料１（第４月目以降）,外来栄養食事指導料,悪性腫瘍特異物質治療管理料（その他・１項目）,心臓ペースメーカー指導管理料（イ以外）,悪性腫瘍特異物質治療管理料（その他・２項目以上）,慢性維持透析患者外来医学管理料,難病外来指導管理料,入院栄養食事指導料,集団栄養食事指導料,療養費同意書交付料,肺血栓塞栓症予防管理料,退院時共同指導料２,診療情報提供料（２）,多機関共同指導加算,薬剤管理指導料１（救命救急入院料等算定患者）,薬剤管理指導料（安全管理を要する医薬品投与患者）,医療機器安全管理料（生命維持管理装置使用）,介護支援等連携指導料,移植後患者指導管理料（臓器移植後）,糖尿病透析予防指導管理料,入院栄養食事指導料１,外来栄養食事指導料１（初回）,外来栄養食事指導料１（２回目以降）（対面）,入院栄養食事指導料１（初回）,入院栄養食事指導料１（２回目）,遠隔モニタリング加算（心臓ペースメーカー指導管理料）,手帳記載加算（薬剤情報提供料）,退院時薬剤情報管理指導料,在宅自己腹膜灌流指導管理料,在宅自己連続携行式腹膜灌流頻回指導管理,在宅酸素療法指導管理料（その他）,在宅自己導尿指導管理料,注入器加算,在宅人工呼吸指導管理料,血糖自己測定器加算（２０回以上）（１型糖尿病の患者を除く）,血糖自己測定器加算（４０回以上）（１型糖尿病の患者を除く）,酸素濃縮装置加算,酸素ボンベ加算（携帯用酸素ボンベ）,紫外線殺菌器加算,自動腹膜灌流装置加算,血糖自己測定器加算（６０回以上）（１型糖尿病の患者を除く）,訪問看護指示料,特別訪問看護指示加算,間歇導尿用ディスポーザブルカテーテル加算,在宅自己注射指導管理料（１以外の場合）,人工呼吸器加算（人工呼吸器）,在宅持続陽圧呼吸療法指導管理料,血糖自己測定器加算（４０回以上）（１型糖尿病・小児低血糖症等）,血糖自己測定器加算（６０回以上）（１型糖尿病・小児低血糖症等）,血糖自己測定器加算（９０回以上）（１型糖尿病・小児低血糖症等）,注入器用注射針加算（１型糖尿病、血友病患者又はこれに準ずる患者）,注入器用注射針加算（その他）,排痰補助装置加算,在宅自己注射指導管理料（１以外の場合）（月２８回以上）,導入初期加算（在宅自己注射指導管理料）,在宅自己注射指導管理料（１以外の場合）（月２７回以下）,在宅持続陽圧呼吸療法指導管理料２,特殊カテーテル加算（間歇導尿用カテーテル）（イ以外）,在宅持続陽圧呼吸療法用治療器加算（ＣＰＡＰを使用）,在宅酸素療法材料加算（その他）,麻薬等加算（調剤料）（入院外）,麻薬等加算（調剤料）（入院）,調剤料（内服薬・浸煎薬・屯服薬）,調剤料（外用薬）,調剤料（入院）,処方料（その他）,麻薬等加算（処方料）,薬剤管理指導料（１の患者以外の患者）,調基（入院）,調基（その他）,薬剤情報提供料,処方料（７種類以上内服薬）,処方箋料（７種類以上内服薬）,処方箋料（その他）,抗悪性腫瘍剤処方管理加算（処方料）,抗悪性腫瘍剤処方管理加算（処方箋料）,処方箋料（向精神薬多剤投与）,生物学的製剤注射加算,精密持続点滴注射加算,麻薬注射加算,皮内、皮下及び筋肉内注射,静脈内注射,点滴注射,中心静脈注射,中心静脈注射用カテーテル挿入,関節腔内注射,動脈注射（その他）,気管内注入,無菌製剤処理料２,植込型カテーテルによる中心静脈注射,中心静脈注射用植込型カテーテル設置（頭頸部その他）,点滴注射（その他）（入院外）,血漿成分製剤加算（点滴注射）,血漿成分製剤加算（中心静脈注射）,無菌製剤処理料１（イ以外）,末梢留置型中心静脈注射用カテーテル挿入,カフ型緊急時ブラッドアクセス用留置カテーテル挿入,硝子体内注射,外来化学療法加算１（抗悪性腫瘍剤以外の薬剤・１５歳以上）,無菌製剤処理料１（閉鎖式接続器具使用）（揮発性の高い薬剤）,外来化学療法加算１（抗悪性腫瘍剤・１５歳以上）,無菌製剤処理料１（閉鎖式接続器具使用）,時間外加算２（イに該当を除く）（処置）,休日加算２（イに該当を除く）（処置）,深夜加算２（イに該当を除く）（処置）,創傷処置（１００ｃｍ２未満）,創傷処置（１００ｃｍ２以上５００ｃｍ２未満）,創傷処置（３０００ｃｍ２以上６０００ｃｍ２未満）,胸腔穿刺（洗浄、注入及び排液を含む）,胸腔試験穿刺,腹腔穿刺（人工気腹、洗浄、注入及び排液を含む）,経皮的肝膿瘍等穿刺術,喀痰吸引,内視鏡下気管支分泌物吸引,持続的胸腔ドレナージ,胃持続ドレナージ,酸素吸入,酸素テント,間歇的陽圧吸入法,高気圧酸素治療（救急的・１人用）,イレウス用ロングチューブ挿入法,人工腎臓（その他）,時間外・休日加算（人工腎臓）,人工腎臓（導入期）加算,血漿交換療法,吸着式血液浄化法,連続携行式腹膜灌流,腹膜灌流（その他）,救命のための気管内挿管,人工呼吸,ＣＰＡＰ,非開胸的心マッサージ,カウンターショック（その他）,心膜穿刺,胃洗浄,皮膚科軟膏処置（１００ｃｍ２以上５００ｃｍ２未満）,膀胱洗浄,膀胱洗浄及び薬液膀胱内注入（カテーテル留置中）,留置カテーテル設置,耳垢栓塞除去（複雑）（片）,ネブライザー,超音波ネブライザー,関節穿刺（片）（処置）,鼻腔栄養,人工呼吸（５時間超）,骨髄穿刺（その他）（処置）,腎嚢胞穿刺（処置）,皮膚科光線療法（長波・中波紫外線、２９０ｎｍ以上３１５ｎｍ以下）,持続緩徐式血液濾過,ドレーン法（ドレナージ）（持続的吸引）,ドレーン法（ドレナージ）（その他）,障害者等加算（人工腎臓）,人工腎臓（慢性維持透析）（４時間未満）,間歇的導尿,摘便,鶏眼・胼胝処置,血球成分除去療法,人工呼吸（鼻マスク式人工呼吸器）,人工呼吸（鼻マスク式人工呼吸器）（５時間超）,四肢ギプスシーネ（手指及び手、足）（片）,重度褥瘡処置（１００ｃｍ２未満）,扁桃処置,人工腎臓（慢性維持透析）（４時間以上５時間未満）,人工腎臓（慢性維持透析）（５時間以上）,尿路ストーマカテーテル交換法,カウンターショック（非医療従事者向け自動除細動器を用いた場合）,局所陰圧閉鎖処置（入院）（１００ｃｍ２未満）,局所陰圧閉鎖処置（入院）（１００ｃｍ２以上）,局所陰圧閉鎖処置（入院）（２００ｃｍ２以上）,初回加算（局所陰圧閉鎖処置）（入院）（１００ｃｍ２未満）,初回加算（局所陰圧閉鎖処置）（入院）（２００ｃｍ２以上）,透析液水質確保加算１,透析液水質確保加算２,障害者等加算（持続緩徐式血液濾過）,ハイフローセラピー,ハイフローセラピー（１５歳以上）,人工腎臓（慢性維持透析１）（４時間未満）（規定患者）,人工腎臓（慢性維持透析１）（４時間以上５時間未満）（規定患者）,人工腎臓（慢性維持透析１）（５時間以上）（規定患者）,導入期加算１（人工腎臓）,導入期加算２（人工腎臓）,透析液水質確保加算（人工腎臓）,創傷処理（筋肉、臓器に達しない）（長径５ｃｍ未満）,創傷処理（筋肉、臓器に達しない）（長径５ｃｍ以上１０ｃｍ未満）,創傷処理（筋肉、臓器に達する）（長径５ｃｍ未満）,創傷処理（筋肉、臓器に達する）（長径５ｃｍ以上１０ｃｍ未満）,皮膚切開術（長径１０ｃｍ未満）,皮膚切開術（長径１０ｃｍ以上２０ｃｍ未満）,皮膚皮下粘膜下血管腫摘出術（露出部、長径３ｃｍ〜６ｃｍ未満）,腐骨摘出術（足その他）,四肢切断術（大腿）,四肢切断術（下腿）,四肢切断術（指）,化膿性又は結核性関節炎掻爬術（肩）,断端形成術（軟部形成のみ）（指）,断端形成術（骨形成を要する）（指）,脊椎骨掻爬術,脊椎骨（軟骨）組織採取術（試験切除）（その他）,結膜結石除去術（少数）,虹彩光凝固術,網膜復位術,硝子体茎顕微鏡下離断術（その他）,鼓膜切開術,鼻腔粘膜焼灼術,気管切開術,血管結紮術（その他）,内シャント血栓除去術,動脈形成術、吻合術（その他の動脈）,内シャント設置術,外シャント設置術,血管移植術、バイパス移植術（その他の動脈）,四肢の血管拡張術・血栓除去術,リンパ節摘出術（長径３ｃｍ未満）,試験開腹術,急性汎発性腹膜炎手術,大網、腸間膜、後腹膜腫瘍摘出術（腸切除を伴わない）,内視鏡的消化管止血術,内視鏡的食道及び胃内異物摘出術,経皮的胆管ドレナージ術,人工肛門造設術,腎摘出術,経皮的腎（腎盂）瘻造設術,保存血液輸血（１回目）,血液型加算（ＡＢＯ式及びＲｈ式）,不規則抗体加算,血液交叉試験加算,間接クームス検査加算,脊椎麻酔,上肢伝達麻酔,球後麻酔,閉鎖循環式全身麻酔５,後頭神経ブロック（局所麻酔剤又はボツリヌス毒素）,網膜光凝固術（通常）,網膜光凝固術（その他特殊）,硬膜外麻酔後における局所麻酔剤の持続的注入,下肢伝達麻酔,増殖性硝子体網膜症手術,水晶体再建術（眼内レンズを挿入）（その他）,内視鏡的胆道ステント留置術,精密持続注入加算（硬膜外麻酔後における局所麻酔剤の持続的注入）,皮膚悪性腫瘍切除術（単純切除）,小腸結腸内視鏡的止血術,精巣悪性腫瘍手術,体外ペースメーキング術,２以上の手術の５０％併施加算,腹腔鏡下脾摘出術,皮膚、皮下腫瘍摘出術（露出部）（長径４ｃｍ以上）,椎間板摘出術（経皮的髄核摘出術）,硝子体茎顕微鏡下離断術（網膜付着組織を含む）,麻酔管理料１（閉鎖循環式全身麻酔）,後発白内障手術,創傷処理（筋肉、臓器に達する）（長径１０ｃｍ以上）,創傷処理（筋肉、臓器に達しない）（長径１０ｃｍ以上）,内視鏡的大腸ポリープ・粘膜切除術（長径２ｃｍ未満）,保存血液輸血（２回目以降）,超音波凝固切開装置等加算,骨移植術（軟骨移植術を含む、自家骨移植）,院内感染防止措置加算（手術）,経尿道的尿管ステント留置術,経尿道的尿管ステント抜去術,上腕動脈表在化法,内視鏡下椎間板摘出（切除）術（後方摘出術）,連続携行式腹膜灌流用カテーテル腹腔内留置術,輸血管理料１,輸血管理料２,閉鎖循環式全身麻酔５（麻酔困難な患者）,閉鎖循環式全身麻酔２（麻酔困難な患者）,閉鎖循環式全身麻酔３（麻酔困難な患者）,閉鎖循環式全身麻酔３,閉鎖循環式全身麻酔４（麻酔困難な患者）,閉鎖循環式全身麻酔４,全層植皮術（２５ｃｍ２未満）,全層植皮術（２５ｃｍ２以上１００ｃｍ２未満）,全層植皮術（１００ｃｍ２以上２００ｃｍ２未満）,画像等手術支援加算（ナビゲーション）,自動縫合器加算,麻酔管理料２（脊椎麻酔）,麻酔管理料２（閉鎖循環式全身麻酔）,脊椎固定術、椎弓切除術、椎弓形成術（椎弓切除）,血管塞栓術（頭部、胸腔、腹腔内血管等）（止血術）,血管塞栓術（頭部、胸腔、腹腔内血管等）（その他）,経皮的シャント拡張術・血栓除去術,腹腔鏡下試験開腹術,腹腔鏡下試験切除術,内視鏡的胆道結石除去術（その他）,膀胱悪性腫瘍手術（経尿道的手術）（電解質溶液利用）,人工肛門・人工膀胱造設術前処置加算,経皮的冠動脈ステント留置術（その他）,神経ブロック併施加算（イ以外）,流産手術（妊娠１１週まで）（手動真空吸引法）,検査逓減,時間外緊急院内検査加算,尿一般,尿蛋白,糖（試験紙法）,尿グルコース,尿浸透圧,ＮＡＧ（尿）,アルブミン定量（尿）,Ｂｅｎｃｅ　Ｊｏｎｅｓ蛋白同定（尿）,尿沈渣（鏡検法）,糞便塗抹,虫卵検出（集卵法）（糞便）,糞便中ヘモグロビン定性,クロストリジオイデス・ディフィシル抗原定性,ＥＳＲ,レチクロ,末梢血液一般検査,血液浸透圧,末梢血液像（鏡検法）,ＨｂＡ１ｃ,骨髄像,全血凝固,ＰＴ,ＡＰＴＴ,フィブリノゲン半定量,ＦＤＰ定性,ＶＷＦ活性,第８凝固因子インヒビター,ＰＩＶＫＡ−２,プラスミノゲン活性,ＰＩＣ,凝固因子（第２因子）,凝固因子（第５因子）,凝固因子（第７因子）,凝固因子（第８因子）,凝固因子（第９因子）,凝固因子（第１３因子）,ＢＩＬ／総,ＢＩＬ／直,ＴＰ,ＺＴＴ,ＴＴＴ,Ａｌｂ（ＢＣＰ改良法・ＢＣＧ法）,ＢＵＮ,クレアチニン,ＵＡ,グルコース,ＬＤ,ＡＬＰ,ＣｈＥ,Ａｍｙ,γ−ＧＴ,ＬＡＰ,ＣＫ,ＡＬＤ,ＴＧ,ナトリウム及びクロール,カリウム,カルシウム,イオン化カルシウム,無機リン及びリン酸,Ｆｅ,マグネシウム,Ｔｃｈｏ,ＡＳＴ,ＡＬＴ,蛋白分画,リン脂質,ＨＤＬ−コレステロール,ＴＩＢＣ（比色法）,ＵＩＢＣ（比色法）,リパーゼ,重炭酸塩,Ｃｕ,ＡＤＡ,グアナーゼ,乳酸,ピルビン酸,アンモニア,ＴＢＡ,ＡＬＰアイソザイム,アミラーゼアイソザイム,ＡＳＴアイソ,ＣＫアイソ,ＬＤアイソ,リポ蛋白分画,ケトン体分画,血液ガス分析,セルロプラスミン,ガラクトース,ＴＩＢＣ（ＲＩＡ法）,ＵＩＢＣ（ＲＩＡ法）,トリプシン,ＡＬ,Ｚｎ,ＰＨ,ＡＣＥ,ビタミンＢ１２,ビタミンＢ２,ビタミンＣ,ＨＣＧ定性,Ｔ３,ＩＲＩ,ＴＳＨ,Ｔ４,ＧＨ,ガストリン,レニン活性,ＰＲＬ,ＨＣＧ半定量,ＨＣＧ−β,ＦＳＨ,ＣＰＲ,ＦＴ３,ＦＴ４,カテコールアミン分画,カルシトニン,テストステロン,コルチゾール,アルドステロン,サイログロブリン,プロゲステロン,メタネフリン・ノルメタネフリン分画,Ｅ２,ｃＡＭＰ,ＰＴＨ,ＡＣＴＨ,ソマトメジンＣ,ＴＲＡｂ,ＡＤＨ,ＣＥＡ,β２−マイクログロブリン,ＡＦＰ,フェリチン半定量,ＤＵＰＡＮ−２,ＣＡ１９−９,ＣＡ１５−３,ＳＣＣ抗原,ＰＳＡ,ＮＳＥ,ＣＡ１２５,結石分析,脂肪酸分画,アミノ酸,ＡＢＯ,Ｒｈ（Ｄ）,Ｃｏｏｍｂｓ試験（直接）,Ｃｏｏｍｂｓ試験（間接）,抗血小板抗体,ＳＴＳ定性,ＡＳＯ定性,ＳＴＳ定量,ＡＳＫ定性,梅毒トレポネーマ抗体定性,マイコプラズマ抗体定性,アデノウイルス抗体価（定性・半定量・定量）,コクサッキーウイルス抗体価（定性・半定量・定量）,エコーウイルス抗体価（定性・半定量・定量）,ヘルペスウイルス抗体価（定性・半定量・定量）,インフルエンザウイルスＡ型抗体価（定性・半定量・定量）,インフルエンザウイルスＢ型抗体価（定性・半定量・定量）,ＲＳウイルス抗体価（定性・半定量・定量）,風疹ウイルス抗体価（定性・半定量・定量）,トキソプラズマ抗体,百日咳菌抗体半定量,Ａ群β溶連菌迅速試験定性,ＨＴＬＶ−１抗体,グロブリンクラス別ウイルス抗体価（ヘルペス）,グロブリンクラス別ウイルス抗体価（風疹）,ＨＢｓ抗体半定量,ＨＢｓ抗原,ＨＢｓ抗体,ＨＢｅ抗原,ＨＢｅ抗体,寒冷凝集,ＲＦ半定量,抗サイログロブリン抗体半定量,ＬＥテスト定性,抗インスリン抗体,抗ＤＮＡ抗体定性,抗ミトコンドリア抗体定性,ＣＲＰ,クリオグロブリン定性,ＣＨ５０,ＩｇＡ,ＩｇＤ,ＩｇＧ,ＩｇＭ,トランスサイレチン（プレアルブミン）,特異的ＩｇＥ半定量・定量,ハプトグロビン,ＩＥＰ,ＬＳＴ,Ｔ細胞サブセット検査,Ｔ細胞・Ｂ細胞百分率,造血器腫瘍細胞抗原検査,Ｓ−Ｍ,細菌培養同定検査（口腔）,細菌培養同定検査（消化管）,細菌培養同定検査（泌尿器）,細菌培養同定検査（血液）,細菌培養同定検査（その他）,細菌培養同定検査（簡易培養）,嫌気性培養加算（細菌培養同定検査）,抗酸菌同定,抗酸菌薬剤感受性検査,Ｔ−Ｍ（組織切片）,電子顕微鏡病理組織標本作製,免疫染色病理組織標本作製（その他）,細胞診（婦人科材料等）,細胞診（穿刺吸引細胞診、体腔洗浄等）,染色体検査,髄液一般検査,尿・糞便等検査判断料,血液学的検査判断料,生化学的検査（１）判断料,生化学的検査（２）判断料,免疫学的検査判断料,微生物学的検査判断料,病理判断料,肺気量分画測定,フローボリューム,機能的残気量測定,呼気ガス分析,クロージングボリューム測定,肺拡散能力検査,死腔量測定,ＢＭＲ,心カテ（左心）,心カテ（右心）,冠動脈造影加算,体液量測定,血流量測定,心拍出量測定,ＥＣＧ１２,ホルター型心電図検査（８時間超）,体表面心電図,トレッドミルによる負荷心肺機能検査,サイクルエルゴメーターによる心肺機能検査,脈波図、心機図、ポリグラフ検査（３又は４検査）,超音波検査（Ａモード法）,超音波検査（断層撮影法）（胸腹部）,パルスドプラ法加算,超音波検査（心臓超音波検査以外）（断層撮影法とＭモード法）,超音波検査（心臓超音波検査）（経胸壁心エコー法）,呼吸心拍監視,観血的動脈圧測定（１時間超）,中心静脈圧測定（４回以下）,中心静脈圧測定（５回以上）,ＥＥＧ８,聴性誘発反応検査,誘発筋電図,尿流,標準純音聴力検査,鼻腔通気度検査,チンパノメトリー,精密眼底検査（片）,精密眼底検査（両）,眼底カメラ,眼底カメラ撮影（蛍光眼底法）,スリットＭ（前・後眼部）,動的量的視野検査（片）,動的量的視野検査（両）,静的量的視野検査（片）,屈折,屈折検査（薬剤使用前後）（６歳未満）,矯正視力検査（眼鏡処方箋の交付）,精密眼圧,角膜曲率,定量的色盲表検査,精密眼筋及び輻輳,眼球突出度測定,スリットＭ（前眼部）,スリットＭ（前眼部）後生体染色使用再検査,涙液分泌機能検査,涙管通水・通色素検査,肝クリアランステスト,腎クリアランステスト,耐糖能精密検査,皮内反応検査（２１箇所以内）,甲状腺ラジオアイソトープ摂取率（ＲＩ）,骨塩定量検査（ＤＥＸＡ法による腰椎撮影）,ＥＦ−嗅裂・鼻咽腔・副鼻腔,ＥＦ−喉頭,ＥＦ−気管支,胸腔鏡検査,ＥＦ−胃・十二指腸,ＥＦ−直腸,大腸内視鏡検査（ファイバースコピー・Ｓ状結腸）,大腸内視鏡検査（ファイバースコピー・下行結腸及び横行結腸）,大腸内視鏡検査（ファイバースコピー・上行結腸及び盲腸）,ＥＦ−腹腔,ＥＦ−膀胱尿道,Ｂ−Ｖ,腰椎穿刺（脳脊髄圧測定を含む）,骨髄穿刺（その他）（検査）,関節穿刺（片）（検査）,リンパ節等穿刺又は針生検,経皮的針生検法,内視鏡下生検法,組織試験採取、切採法（皮膚）,組織試験採取、切採法（鼻）,子宮頸管粘液採取,子宮腟部組織採取,子宮内膜組織採取,胸水採取,Ｂ−Ａ,アミノ酸（５種類以上）,呼吸心拍監視（３時間超）（７日以内）,肺炎球菌抗原定性（尿）,グロブリンクラス別ウイルス抗体価（サイトメガロ）,先天性代謝異常症スクリーニングテスト（尿）,ロタウイルス抗原定性（糞便）,ＡＴ活性,α１−ＡＴ,Ｄダイマー定性,プロテインＣ活性,ＴＡＴ,リポ蛋白分画（ＰＡＧディスク電気泳動法）,ＣＫ−ＭＢ（免疫阻害法・蛋白量測定）,葉酸,エンドトキシン,レニン定量,遊離テストステロン,ＡＮＰ,ＮＣＣ−ＳＴ−４３９,ＣＡ７２−４,ＳＰａｎ−１,ＰＩＶＫＡ−２半定量,ＳＬＸ,ＲＳウイルス抗原定性,グロブリンクラス別クラミジア・トラコマチス抗体,グロブリンクラス別ウイルス抗体価,ＨＩＶ−１抗体（ウエスタンブロット法）,抗ＡＣｈＲ抗体,ＨＣＶ抗体定性・定量,非特異的ＩｇＥ半定量,Ｃ１ｑ結合免疫複合体,終夜睡眠ポリグラフィー（携帯用装置使用）,下垂体前葉負荷試験（ＡＣＴＨ）,副腎皮質負荷試験（鉱質コルチコイド）,副腎皮質負荷試験（糖質コルチコイド）,甲状腺穿刺又は針生検,ＨＢｃ抗体半定量・定量,ＨＡ抗体,ＨＡ−ＩｇＭ抗体,ＨＢｃ−ＩｇＭ抗体,抗核抗体（蛍光抗体法除く。）,凝固因子（第１２因子）,グロブリンクラス別ウイルス抗体価（ＥＢ）,腎嚢胞穿刺（検査）,Ｍｂ定性,ビタミンＢ１,Ｃ３,Ｃ４,Ｔｆ,ＶＷＦ抗原,プロテインＳ活性,４型コラーゲン・７Ｓ,エリスロポエチン,ＢＵＮ（尿）,クレアチン（尿）,クレアチニン（尿）,ＵＡ（尿）,アミラーゼ（尿）,ナトリウム及びクロール（尿）,カリウム（尿）,カルシウム（尿）,マグネシウム（尿）,蛋白分画（尿）,Ｃｕ（尿）,Ｚｎ（尿）,α１−マイクログロブリン（尿）,ＨＣＧ半定量（尿）,ＨＣＧ−β（尿）,ＣＰＲ（尿）,アルドステロン（尿）,アミノ酸定性（尿）,β２−マイクログロブリン（尿）,抗サイログロブリン抗体,カンジダ抗原定性,ＭＡＣ核酸検出,１．５ＡＧ,細菌培養同定検査（気道）,細菌培養同定検査（呼吸器）,細菌培養同定検査（生殖器）,細菌培養同定検査（穿刺液）,腹水採取,４型コラーゲン,基本的検体検査実施料（４週間以内）,抗ＲＮＰ抗体定性,抗Ｓｍ抗体定性,抗ＳＳ−Ａ／Ｒｏ抗体定性,抗Ｓｃｌ−７０抗体定性,抗ＳＳ−Ｂ／Ｌａ抗体定性,細菌薬剤感受性検査（１菌種）,細菌薬剤感受性検査（２菌種）,細菌薬剤感受性検査（３菌種以上）,スリットＭ（前・後眼部）後生体染色使用再検査,呼吸機能検査等判断料,脳波検査判断料２,神経・筋検査判断料,ラジオアイソトープ検査判断料,粘膜点墨法加算（検査）,経気管肺生検法,角膜内皮細胞顕微鏡検査,超音波内視鏡検査加算,アデノウイルス抗原定性（糞便を除く。）,基本的検体検査判断料,ＬＨ,グリコアルブミン,ヒアルロン酸,ＯＣ,トキソプラズマＩｇＭ抗体,クリプトコックス抗原定性,抗Ｊｏ−１抗体定性,ＴｎＴ定性・定量,ＤＨＥＡ−Ｓ,ＨＩＶ−１、２抗体定性,リポ蛋白（ａ）,ＲＬＰ−Ｃ,ＡＮＣＡ定性,抗カルジオリピンβ２グリコプロテインＩ複合体抗体,ＨＩＶ−２抗体（ウエスタンブロット法）,ＰＴＨｒＰ,組織診断料,経皮的動脈血酸素飽和度測定,中心フリッカー試験,トロンボモジュリン,グロブリンクラス別ウイルス抗体価（麻疹）,グロブリンクラス別ウイルス抗体価（ムンプス）,抗甲状腺ペルオキシダーゼ抗体,総分岐鎖アミノ酸／ＢＴＲ,トランスフェリン（尿）,結核菌群核酸検出,ｓＩＬ−２Ｒ,１．２５−ジヒドロキシビタミンＤ３,アスペルギルス抗原,ＨＣＶ核酸定量,クラミジア・トラコマチス核酸検出,シフラ,（１→３）−β−Ｄ−グルカン,ＳＡＡ,ＨＢＶ核酸定量,超音波検査（心臓超音波検査）（経食道心エコー法）,終夜睡眠ポリグラフィー（１及び２以外）,入院時初回加算,抗ＧＡＤ抗体,ＡＦＰ−Ｌ３％,ＰｒｏＧＲＰ,ＢＮＰ,ＨＣＶ血清群別判定,ＴＳＡｂ,ＨＩＶ−１核酸定量,サイトメガロウイルスｐｐ６５抗原定性,抗セントロメア抗体定性,抗カルジオリピン抗体,淋菌核酸検出,ＮＴＸ,大腸菌Ｏ１５７抗原定性,ＤＰＤ（尿）,超音波検査（断層撮影法）（その他）,造血器腫瘍遺伝子検査,基本的検体検査実施料（４週間超）,呼吸心拍監視（７日超１４日以内）,呼吸心拍監視（１４日超）,筋電図（１筋につき）,ＬＤＬ−コレステロール,クラミドフィラ・ニューモニエＩｇＧ抗体,グロブリンクラス別ウイルス抗体価（ヒトパルボウイルスＢ１９）,ＨＣＶコア蛋白,ＭＰＯ−ＡＮＣＡ,ＳＰ−Ｄ,ＫＬ−６,ＢＡＰ,抗ガラクトース欠損ＩｇＧ抗体定性,ＮＭＰ２２定性（尿）,ＰＳＡ　Ｆ／Ｔ比,抗ＧＢＭ抗体,ループスアンチコアグラント定性,インフルエンザウイルス抗原定性,抗酸菌分離培養検査（それ以外）,検体検査管理加算（１）,残尿測定検査（超音波によるもの）,耳音響放射（ＯＡＥ）検査（その他）,汎網膜硝子体検査（片）,角膜形状解析検査,ヘリコバクター・ピロリ抗体定性・半定量,ＵＢＴ,ＭＭＰ−３,抗ＬＫＭ−１抗体,ＨＥＲ２タンパク,抗酸菌抗体定性,水痘・帯状疱疹ウイルス抗体価（定性・半定量・定量）,レジオネラ抗原定性（尿）,抗デスモグレイン１抗体,ヘリコバクター・ピロリ抗原定性,赤血球・好中球表面抗原検査,簡易聴力検査（気導純音聴力）,クレアチニン試験紙法（尿）（蛋白／クレアチニン比）,抗ＩＡ−２抗体,クラミドフィラ・ニューモニエＩｇＭ抗体,シスタチンＣ,ペントシジン,結核菌特異的インターフェロン−γ産生能,ＰＣＴ半定量,外来迅速検体検査加算,ＩｇＧインデックス,オリゴクローナルバンド,免疫関連遺伝子再構成,１ＣＴＰ,ＰＡ−ＩｇＧ,組織診断料（他医療機関作製の組織標本）,矯正視力検査（１以外）,イヌリン,抗シトルリン化ペプチド抗体定性,ＮＴ−ｐｒｏＢＮＰ,ｕｃＯＣ,抗ＢＰ１８０−ＮＣ１６ａ抗体,ＷＴ１　ｍＲＮＡ,抗核抗体（蛍光抗体法）定性,造影剤使用加算（超音波検査）,２４時間自由行動下血圧測定,眼底三次元画像解析,小腸内視鏡検査（カプセル型内視鏡）,組織試験採取、切採法（末梢神経）,ＭＤＡ−ＬＤＬ,ＴＡＲＣ,ＴＲＡＣＰ−５ｂ,４種類以上抗体使用加算,細胞診断料,ＩｇＧ４,ＨＰＶ核酸検出,検体検査管理加算（４）,連続呼気ガス分析加算,残尿測定検査（導尿によるもの）,大腿骨同時撮影加算（ＤＥＸＡ法）,狭帯域光強調加算（検査）,骨髄生検,組織試験採取、切採法（心筋）,抗ＲＮＡポリメラーゼ３抗体,Ｉｎｔａｃｔ　Ｐ１ＮＰ,ＨＢＶジェノタイプ判定,Ｌ−ＦＡＢＰ（尿）,免疫グロブリン遊離Ｌ鎖κ／λ比,ＨＥ−ＩｇＡ抗体定性,病理診断管理加算２（組織診断）,病理診断管理加算２（細胞診断）,末梢血液像（自動機械法）,Ｈ−ＦＡＢＰ定量,梅毒トレポネーマ抗体定量,ＲＦ定量,ＰＲ３−ＡＮＣＡ,骨髄像診断加算,呼吸抵抗測定（広域周波オシレーション法）,呼吸抵抗測定（その他）,加算平均心電図による心室遅延電位測定,超音波検査（心臓超音波検査）（負荷心エコー法）,平衡機能検査（赤外線ＣＣＤカメラ等）,イヌリンクリアランス測定,ガイドシース加算,ＥＢＵＳ−ＴＢＮＡ,血小板第４因子−ヘパリン複合体抗体（ＩｇＧ、ＩｇＭ、ＩｇＡ抗体）,Ｐ１ＮＰ,マイコプラズマ抗原定性（免疫クロマト法）,抗アクアポリン４抗体,抗筋特異的チロシンキナーゼ抗体,プレセプシン定量,抗ＡＲＳ抗体,婦人科材料等液状化検体細胞診加算,抗ミトコンドリア抗体半定量,眼底カメラ撮影（デジタル撮影）,Ｍａｃ−２結合蛋白糖鎖修飾異性体,アポリポ蛋白（１項目）,国際標準検査管理加算,超音波エラストグラフィー,屈折検査（１以外）,鼻腔・咽頭拭い液採取,２５−ヒドロキシビタミンＤ,ＡＤＡＭＴＳ１３活性,ＡＤＡＭＴＳ１３インヒビター,免疫電気泳動法（抗ヒト全血清）,免疫電気泳動法（特異抗血清）,ＬＳＴ（１薬剤）,ＬＳＴ（２薬剤）,ＬＳＴ（３薬剤以上）,ＥＢウイルス核酸定量,超音波検査（断層撮影法）（下肢血管）,悪性腫瘍病理組織標本加算,電子画像管理加算（単純撮影）,透視診断,他医撮影の写真診断（単純撮影・イ）,造影剤使用撮影（アナログ撮影）,ＣＴ撮影（１６列以上６４列未満マルチスライス型機器）,造影剤使用加算（ＣＴ）,造影剤注入手技（動脈造影カテーテル法）（イ以外）,造影剤注入手技（静脈造影カテーテル法）,造影剤注入手技（注腸）,造影剤注入手技（その他）,甲状腺ラジオアイソトープ摂取率測定加算,シングルホトンエミッションコンピューター断層撮影,核医学診断（１以外）,コンピューター断層診断,時間外緊急院内画像診断加算,基本的エックス線診断料（４週間以内）,電子画像管理加算（特殊撮影）,電子画像管理加算（造影剤使用撮影）,他医撮影のコンピューター断層診断,断層撮影負荷試験加算,ＭＲＩ撮影（１．５テスラ以上３テスラ未満の機器）,造影剤使用加算（ＭＲＩ）,基本的エックス線診断料（４週間超）,シンチグラム（部分・静態）,シンチグラム（部分・動態）,シンチグラム（全身）,ポジトロン断層撮影（１８ＦＤＧ使用）,画像診断管理加算１（核医学診断）,画像診断管理加算２（核医学診断）,画像診断管理加算２（コンピューター断層診断）,電子画像管理加算（核医学診断料）,造影剤注入手技（動脈造影カテーテル法）（選択的血管造影）,冠動脈ＣＴ撮影加算,心臓ＭＲＩ撮影加算,単純撮影（デジタル撮影）,特殊撮影（デジタル撮影）,造影剤使用撮影（デジタル撮影）,造影剤注入手技（嚥下造影）,電子画像管理加算（コンピューター断層診断料）,ＣＴ撮影（６４列以上マルチスライス型機器）（その他）,ＭＲＩ撮影（３テスラ以上の機器）（その他）,画像診断管理加算３（核医学診断）,画像診断管理加算３（コンピューター断層診断）,頭部ＭＲＩ撮影加算,傷病手当金意見書交付料,感染症法申請診断書交付料,入院精神療法（２）（６月以内）,診療情報提供料（１）,摂食機能療法（３０分以上）,リハビリテーション総合計画評価料１,医療保護入院等診療料,心大血管疾患リハビリテーション料（１）,脳血管疾患等リハビリテーション料（１）,運動器リハビリテーション料（２）,呼吸器リハビリテーション料（１）,早期リハビリテーション加算,脳血管疾患等リハビリテーション料（１）（廃用症候群）,運動器リハビリテーション料（１）,がん患者リハビリテーション料,初期加算（リハビリテーション料）,廃用症候群リハビリテーション料（１）,退院時リハビリテーション指導料,２級地地域加算,特定機能病院精神病棟１０対１入院基本料,特定機能病院一般病棟入院期間加算（１４日以内）,特定機能病院一般病棟入院期間加算（１５日以上３０日以内）,特定機能病院精神病棟入院期間加算（１４日以内）,特定機能病院精神病棟入院期間加算（１５日以上３０日以内）,特定機能病院精神病棟入院期間加算（３１日以上９０日以内）,救急医療管理加算,診療録管理体制加算２,難病患者等入院診療加算,療養環境加算,重症者等療養環境特別加算（個室）,外泊（入院基本料の減額）,緩和ケア診療加算,基幹型臨床研修病院入院診療加算,ハイケアユニット入院医療管理料（経過措置）,特定機能病院一般病棟７対１入院基本料,３級地地域加算,がん診療連携拠点病院加算,医療安全対策加算１,褥瘡ハイリスク患者ケア加算,ハイリスク分娩管理加算,妊産婦緊急搬送入院加算,二類感染症患者療養環境特別加算（個室）,精神科身体合併症管理加算（７日以内）,ハイリスク妊娠管理加算,５０対１急性期看護補助体制加算,７５対１急性期看護補助体制加算,特定集中治療室管理料４（特定集中治療室管理料・７日以内）,特定集中治療室管理料４（特定集中治療室管理料・８日〜１４日）,看護職員夜間配置加算,無菌治療室管理加算２,精神科リエゾンチーム加算,感染防止対策加算１,感染防止対策地域連携加算,患者サポート体制充実加算,退院調整加算（一般病棟入院等）（〜１４日）,退院調整加算（一般病棟入院等）（１５日〜３０日）,退院調整加算（一般病棟入院等）（３１日〜）,病棟薬剤業務実施加算,データ提出加算２（許可病床数２００床以上）,救急医療管理加算１,救急医療管理加算２,診療録管理体制加算１,１５対１補助体制加算（医師事務作業補助体制加算１）,２０対１補助体制加算（医師事務作業補助体制加算１）,２５対１補助体制加算（医師事務作業補助体制加算１）,３０対１補助体制加算（医師事務作業補助体制加算１）,４０対１補助体制加算（医師事務作業補助体制加算１）,５０対１補助体制加算（医師事務作業補助体制加算１）,精神科身体合併症管理加算（８日以上１５日以内）,ハイケアユニット入院医療管理料１,看護職員夜間１２対１配置加算２,病棟薬剤業務実施加算１,病棟薬剤業務実施加算２,入退院支援加算２（一般病棟入院基本料等）,短手３（経皮的シャント拡張術・血栓除去術）,抗菌薬適正使用支援加算,後発医薬品使用体制加算１,提出データ評価加算,早期離床・リハビリテーション加算（特定集中治療室管理料）,ハイケアユニット入院医療管理料（１４日以内）（経措）,ハイケアユニット入院医療管理料（１５日以上２１日以内）（経措）,特定集中治療室管理料４（特定集中治療室管理料・７日以内）,特定集中治療室管理料４（特定集中治療室管理料・８日〜１４日）,ハイケアユニット入院医療管理料１（１４日以内）,ハイケアユニット入院医療管理料１（１５日以上２１日以内）,入院時食事療養（１）（１食につき）（２以外の食事療養）,特別食加算（食事療養）,食堂加算（食事療養）,食事療養標準負担額（一般）,食事療養標準負担額（低２・９０日以下）,食事療養標準負担額（低２・９０日超）,食事療養標準負担額（低１）,入院時食事療養（１）（１食につき）（流動食のみを提供）,食事療養標準負担額（指定難病・小児慢性特定疾病患者）（一般）,注射用蒸留水　２０ｍＬ,マンモグラフィー用フィルム（１８×２４）,画像記録用フィルム（半切）,ダイレーター,血管造影用シースイントロデューサーセット（選択的導入用）,プラスチックカニューレ型静脈内留置針（標準型）,プラスチックカニューレ型静脈内留置針（針刺し事故防止機構付加型）,中心静脈用カテーテル（標準・シングルルーメン）,ヘモダイアフィルター,冠動脈用ステントセット（再狭窄抑制型）,塞栓用コイル（コイル・水圧式・ワイヤー式デタッチャブル型）,体外式連続心拍出量測定用センサー,ホローファイバー型及び積層型（キール型）（１．５ｍ２未満）（３）,ホローファイバー型及び積層型（キール型）（１．５ｍ２未満）（４）,ホローファイバー型及び積層型（キール型）（１．５ｍ２以上）（４）,肝動脈塞栓材,カプセル型内視鏡（小腸用）,血管内血栓異物除去用留置カテーテル（一般型）,血管内光断層撮影用カテーテル,塞栓用コイル（コイル・特殊型）,皮膚欠損用創傷被覆材（皮下組織に至る創傷用・標準型）,中心静脈注射用カテーテル,局所陰圧閉鎖処置用材料,血管造影用圧センサー付材料（血管造影用圧ガイドワイヤー）,ホローファイバー型及び積層型（キール型）（2．0m2以上）（4）,輸血用血液フィルター（カリウム除去用）,血管内手術用カテーテル（交換用カテーテル）,血管内塞栓材（止血用）,ＰＴＣＡ用カテーテル（再狭窄抑制型）,人工血管（永久留置型・小血管用・特殊型・外部サポートなし）,携帯型ディスポーザブル注入ポンプ（ＰＣＡ型）,携帯型ディスポーザブル注入ポンプ（ＰＣＡ型）,腎瘻・膀胱瘻用材料（穿刺針）,経皮・内視鏡的胆管ドレナージ用材料（経鼻法用ワイヤー）,ダイアライザー（１ａ型（膜面積１．５ｍ２未満））,ダイアライザー（１ａ型）,ダイアライザー（２ａ型）,ガイドワイヤー,ドレナージカテーテル,持続緩徐式血液濾過器（標準型）,輸血用血液フィルター（微小凝集塊除去用）,微線維性コラーゲン,血漿交換用ディスポーザブル選択的血漿成分吸着器（劇症肝炎用以外）,吸着式血液浄化器（エンドトキシン）,真皮欠損用グラフト,血管内超音波プローブ（標準・細径）,血管造影用カテーテル（バルーン型（１））,膀胱留置用ディスポーザブルカテーテル（２管一般（１））,膀胱留置用ディスポーザブルカテーテル（２管一般（２）・標準型）,膀胱留置用ディスポーザブルカテーテル（２管一般（３）・標準型）,膀胱留置用ディスポーザブルカテーテル（特定（２））,食道静脈瘤硬化療法用穿刺針,末梢血管用ステントセット（一般型）,冠動脈狭窄部貫通用カテーテル,合成吸収性癒着防止材（シート型）,経皮的血管形成術用穿刺部止血材料,血管内視鏡カテーテル,白血球吸着用材料（一般用）,ＰＴＣＡ用カテーテル（一般型）,腹膜透析液交換セット（交換キット）,腹膜透析液交換セット（回路・Ｙセット）,腹膜透析液交換セット（回路・ＡＰＤセット）,動脈圧モニターカテ末梢動脈用,サーモダイリューション用カテーテル（標準型）,サーモダイリューション用カテーテル（出量測定有・モニター有）,血管造影用マイクロカテーテル（アプローチ型・ブレード有）,血管造影用マイクロカテーテル（造影能強化型）,血管造影用マイクロカテーテル（デタッチャブルコイル用）,中心静脈用カテーテル（標準・マルチルーメン）,中心静脈用カテーテル（標準・マルチルーメン・セルジンガー型）,套管針カテーテル（シングルルーメン・標準型）,套管針カテーテル（シングルルーメン・細径穿刺針型）,套管針カテーテル（ダブルルーメン）,栄養カテーテル（経鼻用・一般用）,気管内チューブ（カフあり・カフ上部吸引機能なし）,胃管カテーテル（ダブルルーメン・標準型）,吸引留置カテーテル（能動吸引型（心嚢・縦隔穿刺用））,吸引留置カテーテル（能動吸引型・創部用・軟質型）,吸引留置カテーテル（受動吸引型・チューブドレーン・フィルム型）,吸引留置カテーテル（受動吸引型・チューブドレーン・チューブ型）,イレウス用ロングチューブ（標準型・経鼻挿入型）,経皮・内視鏡的胆管ドレナージ用カテ（ワンステップ・ダイレクト）,胆道ステントセット（一般型・一時留置型・ステント）,胆道ステントセット（一般型・一時留置型・デリバリーシステム）,尿管ステントセット（一般型・標準型）,尿管ステントセット（一般型・異物付着防止型）,尿管ステントセット（外瘻用・腎盂留置型・標準型）,気管切開後留置用チューブ（一般・カフ付き・吸引有・一重管）,気管切開後留置用チューブ（輪状甲状膜切開）,緊急時ブラッドアクセス用留置カテーテル（シングル・一般型）,緊急時ブラッドアクセス用留置カテーテル（シングル・交換用）,緊急時ブラッドアクセス用留置カテーテル（ダブル以上・一般型）,緊急時ブラッドアクセス用留置カテーテル（ダブル以上・カフ型）,血漿交換用血漿分離器,血漿交換用血漿成分分離器,腹膜透析用接続チューブ,腹膜透析用カテーテル（長期留置型・補強部あり）,腹膜透析用カテーテル（長期留置型・逆U字型,固定用金属ピン（一般用・標準型）,皮膚欠損用創傷被覆材（真皮に至る創傷用）,皮膚欠損用創傷被覆材（皮下組織に至る創傷用・標準型）,体外ペースメーカー用電極（一時ペーシング型）,体表面ペーシング用電極,ＰＴＡバルーンカテーテル（一般型・特殊型）,血栓除去用カテーテル（バルーン付き・一般型）,血栓除去用カテーテル（バルーン付き・極細型）,血栓除去用カテーテル（経皮的血栓除去用）,塞栓用コイル（コイル・標準型）,塞栓用コイル（コイル・機械式デタッチャブル型）,塞栓用コイル（コイル・電気式デタッチャブル型）,塞栓用コイル（プッシャー）,人工血管（永久留置型・小血管用・標準型・外部サポートあり）,人工血管（永久留置型・小血管用・標準型・外部サポートなし）,胆道結石除去用カテーテル（内視鏡バルーン（トリプルルーメン））,腹膜透析液交換セット（回路・Ｙセット）,腹膜透析液交換セット（回路・ＡＰＤセット）,血管造影用シースイントロデューサーセット（一般用）,血管造影用シースイントロデューサーセット（蛇行血管用）,血管造影用カテーテル（一般用）,血管造影用ガイドワイヤー（一般用）,血管造影用ガイドワイヤー（交換用）,血管造影用ガイドワイヤー（微細血管用）,ＰＴＣＡ用カテーテル用ガイドワイヤー（一般用）,ＰＴＣＡ用カテーテル用ガイドワイヤー（複合・高度狭窄部位）,腎瘻・膀胱瘻用カ材料（腎瘻用・ピッグテイル型）,腎瘻・膀胱瘻用材料（腎瘻用カテーテル・マレコ型）,腎瘻・膀胱瘻用材料（腎瘻用カテーテル・腎盂バルーン型）,廃止738320000,ガイディングカテーテル（冠動脈用）,ガイディングカテーテル（その他血管用）,ガイディングカテーテル（脳血管用・標準型）,液体酸素・定置式液化酸素貯槽（ＣＥ）,検査等で使用したガスの費用,未コード化特定器材コード(777770000),コメントコード（810000000）,nan,他科にて請求済み,他科にて徴収済み,ウ　一旦帰宅し、後刻又は後日検査、画像診断、手術等を受けに来院,ア　吐血、喀血又は重篤な脱水で全身状態不良の状態,ウ　呼吸不全又は心不全で重篤な状態,オ　ショック,カ　重篤な代謝障害（肝不全、腎不全、重症糖尿病等）,ケ　緊急の手術、カテーテル治療等又はｔ−ＰＡ療法を必要とする状態,（ロ）　てんかん患者で抗てんかん剤を投与,（チ）　臓器移植術を受けた患者で免疫抑制剤を投与,（リ）　留意事項通知に規定する患者でシクロスポリンを投与,（ヲ）　留意事項通知に規定する患者でタクロリムス水和物を投与,（ワ）　留意事項通知に規定する患者でトリアゾール系抗真菌剤を投与,退院直後,ア　在宅自己連続携行式腹膜灌流の導入期にあるもの,ウ　腹膜炎の疑い、トンネル感染及び出口感染のあるもの,エ　腹膜の透析効率及び除水効率が著しく低下しているもの,オ　その他医師が特に必要と認めるもの,ア　糖尿病等で１日概ね４回以上自己注射が必要,当月分,翌々月分,翌月分,引き続き入院,エ　移植後リンパ増殖性疾患患者（経過観察目的）,ア　急性腎不全の患者,イ　透析導入期（１月に限る。）の患者,エ　特別な管理が必要（ヌ　麻酔による手術を実施した状態）,ア　障害者基本法にいう障害者で留意事項通知に規定する者,エ　留意事項通知に規定する糖尿病の患者,ケ　出血性消化器病変を有する者,サ　重症感染症に合併しているために入院中の患者,ス　入院中の患者であって腹水・胸水が貯留しているもの,チ　人工呼吸を実施中の患者,ア　一方向から造影して９０％以上の狭窄病変,ス　留意事項通知に規定する腎不全の患者,ネ　透析を行っている患者,悪性リンパ腫が疑われる患者,オ　留意事項通知に規定する患者で抗生物質等を数日間以上投与,先進医療の名称；,人工腎臓導入期　平成　　年　　月　　日,連続携行式腹膜灌流導入期　平成　　年　　月　　日,退　院　平成　　年　　月　　日,時間外　　　月　　日　　時　　分,時間外特例　　　月　　日　　時　　分,休日　　　月　　日,深夜　　　月　　日　　時　　分,算定開始　平成　　年　　月　　日,　平成　　年　　月　　日,動脈血酸素飽和度　　　　％,通算実施回数（　　回）,人工腎臓導入期　令和　　年　　月　　日,連続携行式腹膜灌流導入期　令和　　年　　月　　日,傷病手当金意見書交付　令和　　年　　月　　日,退　院　令和　　年　　月　　日
